# Emerging Nanozyme Strategies for Precision Breast Cancer Treatment

**DOI:** 10.1002/advs.202514085

**Published:** 2025-10-27

**Authors:** Jian Zang, Zhaozhou Ren, Haibo Wang, Xing Yang, Yang Song, Jun Liao, Xiaoying Li

**Affiliations:** ^1^ The first Hospital of China Medical University Liaoning 110001 China; ^2^ Institute of Systems Biomedicine Beijing Key Laboratory of Tumor Systems Biology School of Basic Medical Sciences Peking University Beijing 100191 China; ^3^ Shengjing Hospital of China Medical University Liaoning 110004 China

**Keywords:** breast cancer, clinical translation, nanomedicine, nanozyme, tumor microenvironment

## Abstract

Breast cancer, the most common malignant tumor among women worldwide, poses a significant challenge to public health due to its high incidence and mortality rates. While traditional treatment strategies such as surgery, radiation therapy, chemotherapy and targeted therapy have made significant progress, limitations such as tumor heterogeneity, drug resistance, distant metastasis, and systemic side effects remain urgent issues to address. Nanozymes, a new type of nanomaterial with natural enzyme activity, have provided revolutionary opportunities for the precise treatment of breast cancer. This article aims to comprehensively review the progress of nanozyme applications in breast cancer treatment, focusing on how they can improve immunotherapy by regulating the tumor microenvironment and enhancing the immune response, optimize chemotherapy by improving drug delivery efficiency and overcoming drug resistance, and develop photothermal/photodynamic combination therapy to enhance therapeutic efficacy. In addition, this paper will also analyze in depth the key challenges faced by nanozymes in the clinical translation of breast cancer, such as biocompatibility, targeting efficiency, controllability, and large‐scale production, and look forward to its broad prospects as a new treatment method combined with existing strategies, with the hope of providing new ideas for the precise, efficient, and low‐toxicity treatment of breast cancer in the future.

## Graphical abstract

Different types of nanozymes‐mediated chemotherapy, photothermal therapy, immunotherapy, and multiple combined therapies for the effective treatment of breast cancer.

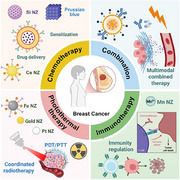

## 1 Introduction

Breast cancer stands as the most frequently diagnosed malignancy among women globally and remains a leading cause of cancer‐related mortality.^[^ ^1^, ^2^, ^3^ ^]^ Recent data from the World Health Organization highlight its escalating prevalence, surpassing lung cancer to become the most common cancer worldwide.^[^ ^4^, ^5^, ^6^ ^]^ Despite substantial advancements in early detection techniques, refined surgical procedures, and sophisticated systemic therapies over the past decades, the overall burden imposed by breast cancer remains significant, manifesting as considerable patient morbidity and profound socioeconomic impact.^[^ ^7^, ^8^, ^9^ ^]^ While improvements in the 5‐year survival rate have been observed across various regions, the long‐term quality of life for survivors is often compromised by treatment‐associated complications and persistent sequelae.^[^ ^10^, ^11^, ^12^ ^]^ Current clinical management protocols for breast cancer typically involve a multimodal approach, integrating surgery, radiotherapy, chemotherapy, endocrine therapy, and targeted therapy.^[^ ^13^, ^14^, ^15^ ^]^ Although these established strategies demonstrate clinical efficacy, they are not without considerable limitations.^[^ ^16^ ^]^ Tumor heterogeneity frequently leads to significant inter‐patient variability in treatment response, complicating therapeutic precision.^[^ ^17^ ^]^ Furthermore, the pervasive development of drug resistance, particularly pronounced in aggressive subtypes like triple‐negative breast cancer (TNBC), often culminates in treatment failure and disease recurrence.^[^ ^18^, ^19^, ^20^ ^]^ Compounding these issues, conventional therapeutic regimens are frequently associated with substantial systemic toxicity and adverse effects, thereby imposing a heavy physical and psychological burden on patients. Consequently, there is an urgent and unmet clinical need to develop more precise, highly efficient, and less toxic therapeutic modalities for breast cancer management.

In this transformative context, the rapid evolution of nanotechnology has unveiled novel avenues for revolutionizing cancer diagnosis and therapy.^[^ ^21^, ^22^, ^23^, ^24^, ^25^, ^26^ ^]^ Among the diverse array of engineered nanomaterials, nanozymes materials endowed with intrinsic enzyme‐like catalytic activities have rapidly emerged as exceptionally promising candidates for advanced biomedical applications.^[^ ^27^, ^28^, ^29^, ^30^, ^31^, ^32^ ^]^ Importantly, breast cancer is particularly suited to nanozyme‐based approaches because of its pronounced molecular heterogeneity, frequent development of multidrug resistance, and the prevalence of hypoxic and immunosuppressive tumor microenvironments. These characteristics create therapeutic bottlenecks for conventional modalities but also provide multiple biochemical targets that can be effectively addressed by the catalytic and multifunctional properties of nanozymes. These sophisticated nanomaterials are capable of mimicking the catalytic functions of their natural enzymatic counterparts, thereby facilitating a myriad of crucial biochemical reactions in vitro and in vivo.^[^ ^33^, ^34^, ^35^, ^36^ ^]^ They exhibit superior stability under challenging physiological conditions, present greater ease of large‐scale synthesis and versatile functionalization, allow for tunable catalytic activity through precise compositional and morphological control, and generally demonstrate favorable biocompatibility. These inherent features strategically position nanozymes as highly versatile tools within the burgeoning field of oncology.^[^ ^37^, ^38^, ^39^, ^40^, ^41^, ^42^, ^43^, ^44^ ^]^ They can be engineered as intelligent drug delivery platforms that transport chemotherapeutic agents, immunomodulators, and photosensitizers directly to tumor sites. This approach increases local drug concentrations within tumors while simultaneously reducing systemic toxicity to healthy organs.^[^ ^45^, ^46^, ^47^ ^]^ On the other hand, the intrinsic catalytic activities of nanozymes, such as their peroxidase (POD)‐like, catalase (CAT)‐like, and superoxide dismutase (SOD)‐like functions, can be strategically harnessed to intricately modulate the tumor microenvironment (TME). For instance, POD‐like nanozymes are capable of catalyzing the efficient conversion of endogenous hydrogen peroxide (H_2_O_2_) into highly cytotoxic hydroxyl radicals (•OH), which in turn promotes severe oxidative damage and induces apoptosis in cancer cells.^[^ ^48^, ^49^, ^50^ ^]^ Conversely, CAT‐like nanozymes can effectively decompose excess H_2_O_2_ to generate molecular oxygen, thereby ameliorating tumor hypoxia, that a notorious impediment to various therapies and consequently enhancing the efficacy of immunotherapies and radiotherapies.^[^ ^51^, ^52^, ^53^, ^54^, ^55^ ^]^ The remarkable multifunctional nature of nanozymes thus bestows them with tremendous potential for achieving synergistic and highly effective cancer treatment.^[^ ^56^, ^57^, ^58^, ^59^ ^]^

**Figure 1:**
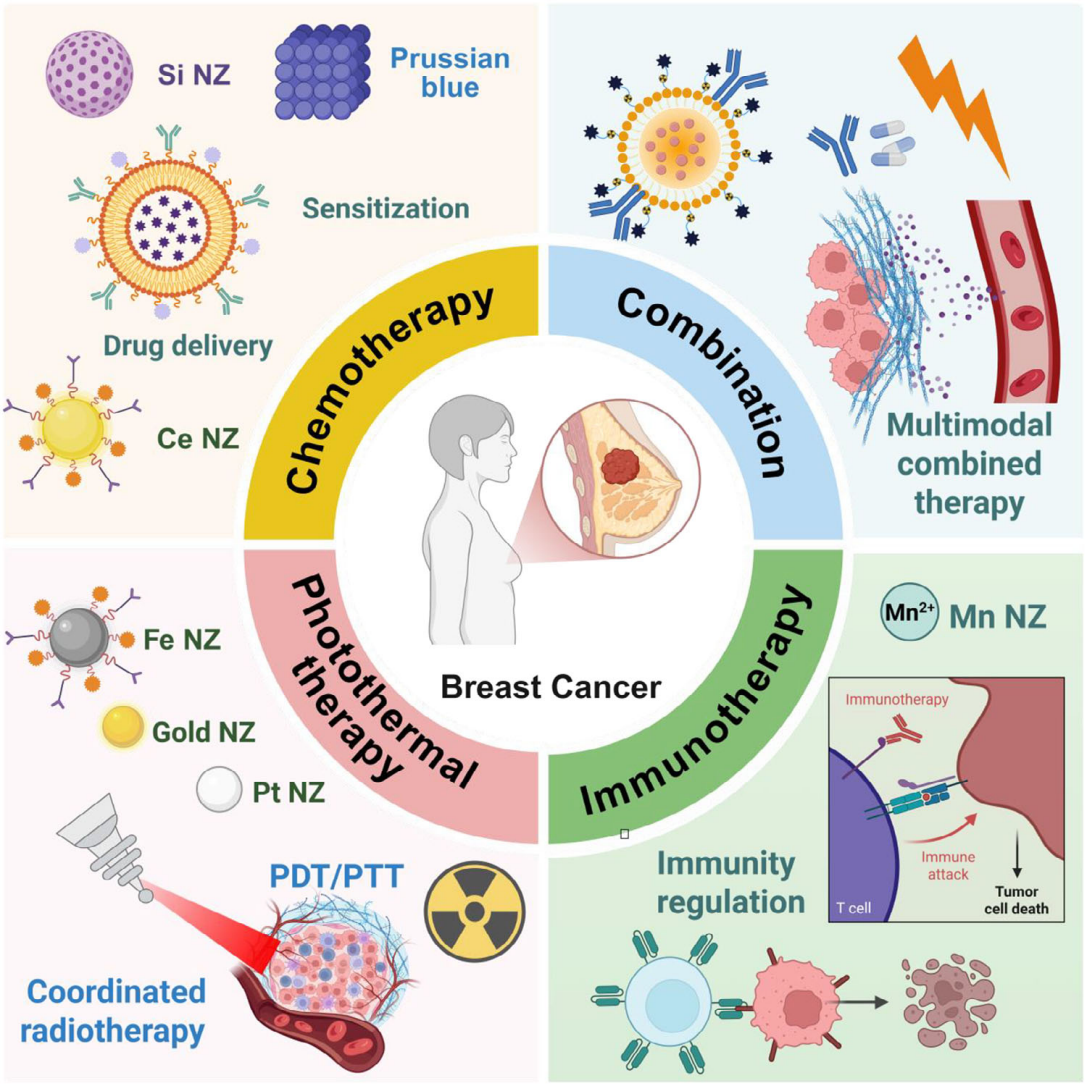
Schematic diagram of nanozyme treatment for breast cancer based on four approaches.

This review aims to provide an exhaustive overview of the most recent advances in nanozyme‐based therapeutic strategies specifically tailored for breast cancer. Our focus extends across four pivotal therapeutic approaches: immunotherapy, chemotherapy, photothermal therapy (PTT), and various combination therapies (**Figure** **1**). For each distinct modality, we meticulously summarize the contemporary design strategies, delineate the underlying therapeutic mechanisms, and present the compelling therapeutic outcomes demonstrated by representative nanozyme platforms. Furthermore, we critically discuss the pervasive current challenges and intrinsic limitations that impede the successful clinical translation of nanozyme‐based systems, including aspects related to biocompatibility, targeting efficiency, controllability, and large‐scale production. Finally, we delineate future perspectives and propose potential directions to accelerate their seamless integration into precision oncology. We sincerely hope that this review serves as an invaluable resource for both researchers and clinicians actively engaged in the fields of nanomedicine and breast cancer research, ultimately contributing to the monumental endeavor of developing safer, more effective, and profoundly impactful treatment options for patients afflicted with breast cancer.

## 2 Molecular Mechanisms Underlying Breast Cancer Pathogenesis

**Figure 2:**
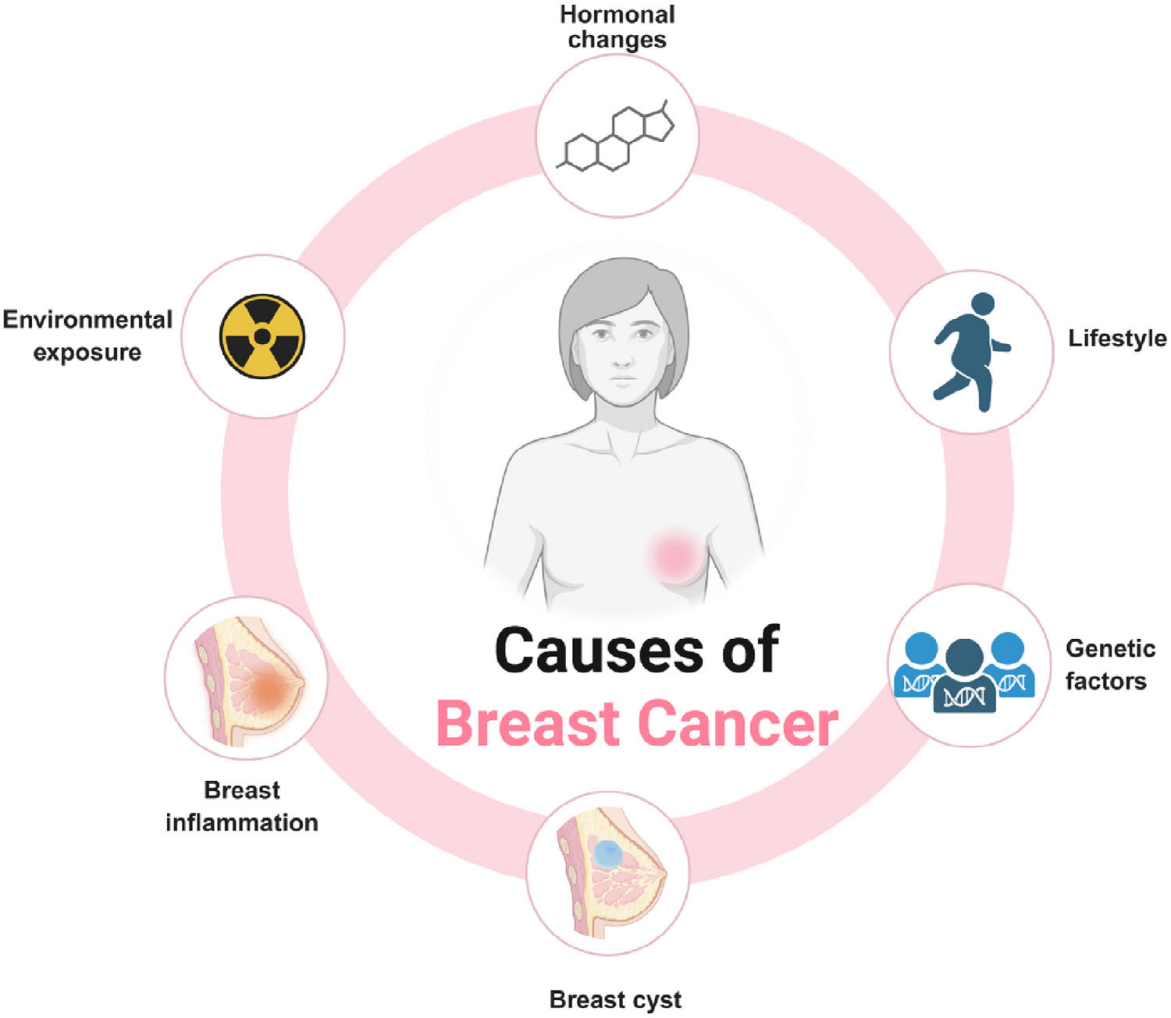
Diagram illustrating the causes of breast cancer.

As a highly heterogeneous disease, the pathogenesis of breast cancer involves complex genetic, epigenetic, and signaling alterations.^[^ ^60^ ^]^ Its causes are diverse, and it is one of the leading causes of cancer‐related deaths. **Figure** **2** illustrates the major pathogenic contributors, including genetic and epigenetic alterations, dysregulated signaling pathways, and microenvironmental influences, which together drive tumor initiation and progression. Despite substantial advancements in diagnosis and therapy, the complex underlying biological processes that drive its initiation, progression, and therapeutic resistance remain areas of intensive investigation.^[^ ^61^ ^]^ A comprehensive understanding of these multifaceted molecular mechanisms is paramount for the development of innovative diagnostic tools and the design of more effective, precise, and less toxic therapeutic strategies. The onset and progression of breast cancer are fundamentally driven by an intricate interplay among intrinsic genetic susceptibility, dynamic epigenetic modifications, dysregulation of pivotal hormonal and intracellular signaling pathways, and the actively involved, evolving TME.

### 2.1 Genetic and Epigenetic Factors

Genetic and epigenetic alterations represent critical hallmarks in the pathogenesis of breast cancer.^[^ ^62^ ^]^ While genetic mutations involve permanent changes to the DNA sequence, epigenetics refers to heritable and reversible modifications in gene expression that occur without altering the underlying DNA sequence.^[^ ^63^ ^]^ These epigenetic modifications, which include DNA methylation, histone modifications, and nucleosome remodeling, are frequently dysregulated in breast cancer, playing a crucial role in its carcinogenic process.

**Figure 3:**
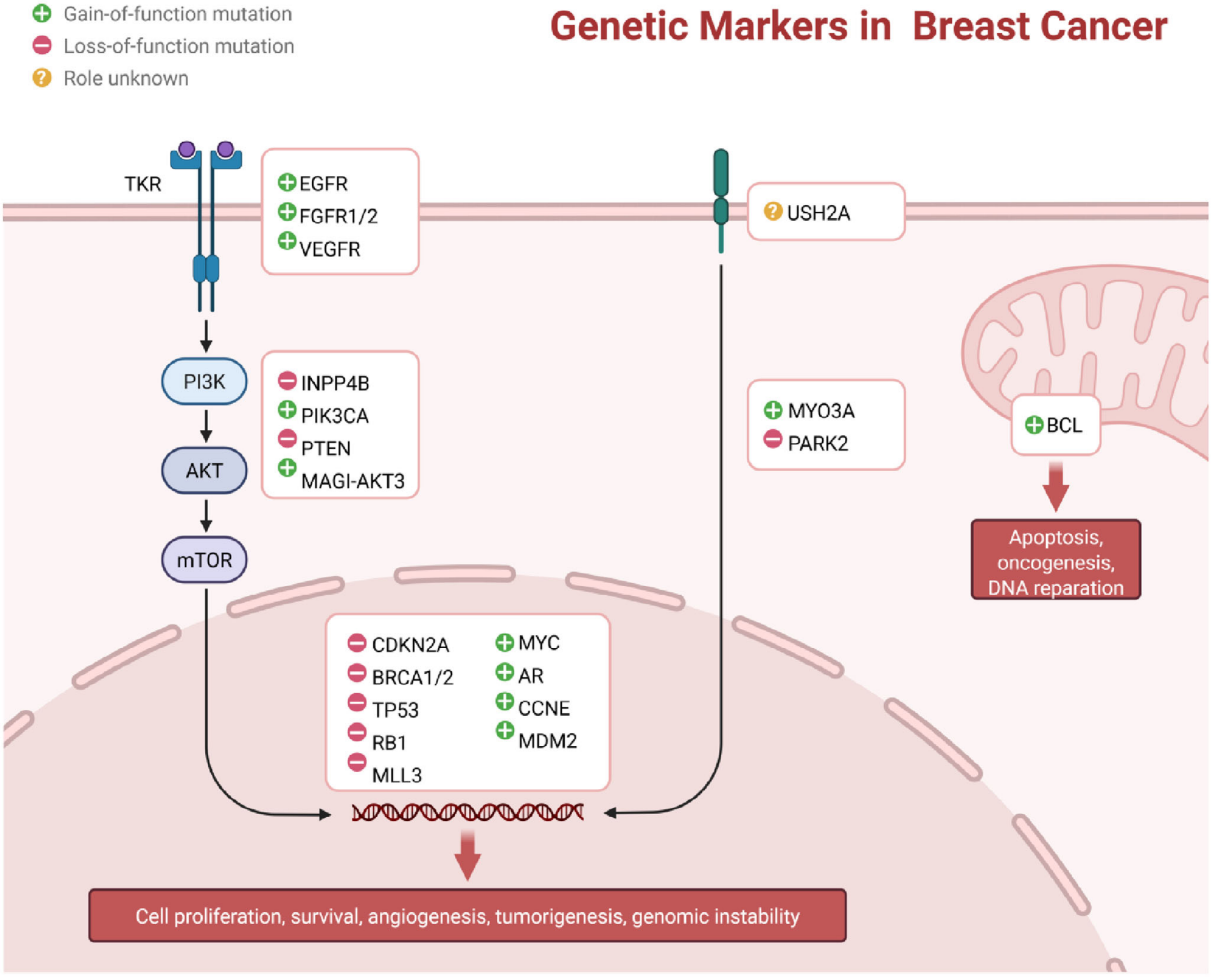
Schematic diagram of genetic markers for breast cancer.

DNA methylation, a key epigenetic modification, commonly involves the addition of a methyl group to the fifth carbon of cytosine residues, predominantly within CpG dinucleotides.^[^ ^64^, ^65^, ^66^ ^]^ In breast cancer cells, common epigenetic alterations include hypermethylation of CpG island regions rich in CpG dinucleotides typically located in gene promoter regions. This hypermethylation leads to the transcriptional silencing of critical tumor suppressor genes and growth regulatory genes, thereby promoting tumorigenesis. The enzymes responsible for this process are DNA methyltransferases (DNMTs), specifically DNMT1, DNMT3a, and DNMT3b, which are often overexpressed in breast cancer. These DNMTs can further interact with histone deacetylases (HDACs) and methyl‐CpG‐binding domain (MBD) proteins to form repressive transcriptional complexes, reinforcing gene silencing.^[^ ^67^, ^68^, ^69^ ^]^ Conversely, widespread hypomethylation of genomic DNA, particularly at oncogene promoters, may also occur in cancer cells, resulting in the increased expression of oncogenes. Promoter hypermethylation is implicated in silencing a diverse array of genes essential for normal cellular function, including estrogen‐responsive genes, pro‐apoptotic genes, cell cycle inhibitor genes, and crucial DNA repair genes such as Breast Cancer Type 1 Susceptibility Gene, BRCA1. Notably, epigenetic silencing mediated by abnormal methylation of the Estrogen Receptor alpha (ERα) promoter is a recognized mechanism underlying the suppression of ERα expression in ER‐negative tumors. This particular epigenetic event can even precede tumor invasion and progressively intensifies with the acquisition of invasiveness and metastatic potential.^[^ ^70^ ^]^ Furthermore, DNA methylation can aberrantly activate the human telomerase reverse transcriptase (hTERT) gene, thereby promoting the immortalization of certain cancer cells. (**Figure** **3**)

Histone modifications, encompassing acetylation, methylation, phosphorylation, and ubiquitination, are dynamic processes that profoundly influence chromatin structure and gene expression.^[^ ^70^, ^71^, ^72^ ^]^ These modifications are catalyzed by a complex interplay of enzymes, including histone acetyltransferases (HATs), histone deacetylases (HDACs), histone methyltransferases (HMTs), and histone demethylases (HDMs). Specifically, histone acetylation of lysine residues within gene promoter regions typically promotes a more open chromatin configuration, rendering DNA accessible for transcription and consequently activating gene expression.^[^ ^73^, ^74^, ^75^ ^]^ Conversely, deacetylation leads to a condensed, transcriptionally silent chromatin state. Distinct histone modification patterns are frequently observed across different breast cancer subtypes; for instance, higher global histone acetylation and methylation levels are often associated with the more prognostically favorable Luminal A subtype, whereas lower levels are characteristic of the aggressive basal‐like subtype. Specific examples include the acetylation of lysine 16 and the loss of trimethylation of lysine 20 in histone H4. Histone methylation patterns exhibit context‐dependent effects, leading to either gene silencing or gene activation.^[^ ^76^ ^]^ The absence of histone H3 lysine 27 trimethylation (H3K27me3) is notably considered a negative prognostic indicator in breast cancer. The intricate function of the estrogen receptor (ER) is also intimately linked to histone modifications, as the E2‐ERα dimer (where E2 represents 17β‐estradiol) actively recruits HATs to remodel chromatin into an open conformation, facilitating gene transcription.^[^ ^77^ ^]^

The core essence of epigenetics lies in its “reversible changes in gene expression,” which fundamentally distinguishes it from irreversible genetic mutations.^[^ ^78^ ^]^ The detailed molecular mechanisms of DNA methylation and histone modifications vividly illustrate how these alterations can silence critical tumor suppressor genes and aberrantly activate oncogenes. Furthermore, the epigenome's dynamic and adaptive nature is significantly influenced by environmental factors, such as exposure to heavy metals. This dynamism suggests that cancer progression is not solely a consequence of fixed genetic errors but is substantially driven by a flexible and mutable epigenetic landscape, shaped by both intrinsic cellular processes and exogenous environmental cues.^[^ ^79^, ^80^, ^81^ ^]^ This inherent reversibility of epigenetic alterations presents a unique and compelling therapeutic opportunity: unlike genetic mutations, epigenetic modifications are potentially amenable to therapeutic intervention. This makes epigenetic therapies, such as HDAC inhibitors and DNMT inhibitors, particularly promising avenues for restoring normal gene function, re‐sensitizing tumors to conventional chemotherapies, and overcoming acquired drug resistance.^[^ ^82^ ^]^ Moreover, this perspective underscores the potential role of lifestyle and environmental interventions in breast cancer prevention or as adjuvant therapies, by favorably modulating the epigenetic landscape.^[^ ^83^ ^]^ Existing data robustly demonstrate that epigenetic changes can silence the BRCA1 gene, paralleling the effects of germline mutations in BRCA1, which are major genetic drivers of breast cancer. Additionally, epigenetic regulation of the ER is a well‐recognized mechanism underlying endocrine resistance in ER‐positive breast cancers.^[^ ^84^ ^]^ This highlights that genetic susceptibility can be mimicked or exacerbated by epigenetic alterations, and critically, epigenetic plasticity can serve as a key mechanism for acquired treatment resistance. These insights reveal that genetic and epigenetic mechanisms are not isolated entities but are deeply interconnected, forming a complex, multi‐layered regulatory network that orchestrates the pathogenesis and therapeutic resistance of breast cancer. Understanding this intricate crosstalk is therefore paramount for designing more effective and durable combination therapies.^[^ ^85^ ^]^ For instance, combining hormone therapy with epigenetic‐targeted therapy may represent a rational strategy to overcome endocrine resistance by restoring ER expression or re‐sensitizing downstream signaling pathways. This integrated perspective is indispensable for the future development of next‐generation precision oncology strategies.

### 2.2 Hormonal and Key Signaling Pathway Dysfunction

The dysregulation of hormonal signaling and other pivotal intracellular pathways is central to breast cancer pathogenesis and resistance development.^[^ ^86^ ^]^ ER and progesterone receptors (PR) are steroid hormone receptors expressed in approximately three‐quarters of breast cancers, classifying them as hormone receptor‐positive (HR+).^[^ ^87^ ^]^ The status of these receptors, typically determined by immunohistochemistry, dictates sensitivity to endocrine therapy. While HR+ tumors generally exhibit a slower growth rate and better short‐term prognosis, they can recur years after initial treatment. Conversely, hormone receptor‐negative (HR‐) cancers, including TNBC, are often more aggressive, lack responsiveness to endocrine therapy, and tend to recur earlier.^[^ ^88^ ^]^

ER signaling occurs through both genomic and non‐genomic actions.^[^ ^89^ ^]^ The classic genomic pathway involves the 17β‐estradiol (E2)‐ER complex translocating to the nucleus, where it directly binds to estrogen response elements (EREs) on DNA or indirectly interacts with other transcription factors.^[^ ^90^ ^]^ This leads to transcriptional activation or silencing of target genes, often involving the recruitment of HATs to open chromatin.^[^ ^91^ ^]^ In contrast, non‐genomic actions are rapid, non‐transcriptional events occurring outside the nucleus, where estrogens bind to membrane‐bound ERs (mbERs) or G protein‐coupled estrogen receptor 1 (GPER1), activating intracellular kinase cascades like the mitogen‐activated protein kinase (MAPK) and phosphatidylinositol 3‐kinase (PI3K)/AKT (serine/threonine kinase)/mammalian target of rapamycin (mTOR) pathways, ultimately impacting cell proliferation and survival.^[^ ^92^, ^93^, ^94^ ^]^ ER activity is also finely tuned by co‐regulatory factors and various post‐translational modifications, which influence its activity, localization, stability, and sensitivity to hormonal responses, thereby contributing to breast cancer progression. Crucially, non‐genomic ER actions can activate key oncogenic pathways like MAPK and PI3K/AKT/mTOR.^[^ ^95^ ^]^ More profoundly, the PI3K/AKT/mTOR pathway itself can activate ER signaling independently of estrogen, a well‐established mechanism associated with endocrine resistance.^[^ ^96^ ^]^ This highlights a clinically relevant and complex crosstalk between these pathways, indicating that breast cancer progression and treatment resistance are driven not by isolated pathways but by an interconnected, adaptive signaling network.^[^ ^97^ ^]^ This underscores the necessity for combination therapies that simultaneously target multiple dysregulated pathways to overcome resistance and achieve more durable antitumor responses.

The PI3K/AKT/mTOR pathway is a critical intracellular signaling network governing fundamental cellular processes such as cell growth, proliferation, metabolism, and survival.^[^ ^98^, ^99^, ^100^ ^]^ Its frequent overactivation is observed in a substantial proportion of breast cancers (25–40%), often due to diverse mechanisms including increased PI3K activity, loss of inhibitory function, or mutations in tumor suppressor genes like inositol polyphosphate 4‐phosphatase type II (INPP4B) and phosphatase and tensin homolog (PTEN).^[^ ^101^ ^]^ The pathway is typically initiated by upstream receptor tyrosine kinases (RTKs), which activate PI3K, a lipid kinase. PI3K phosphorylates phosphatidylinositol 4,5‐bisphosphate (PIP2) to phosphatidylinositol 3,4,5‐trisphosphate (PIP3). The PIK3CA gene, encoding the PI3K p110α catalytic subunit, is the most frequently altered gene in this pathway in breast cancer, with hotspot mutations leading to constitutive PI3K activation observed across various subtypes.^[^ ^102^ ^]^ PIP3 subsequently mediates the phosphorylation and activation of AKT (also known as Protein Kinase B), which profoundly influences cell cycle progression, survival, and growth. Activated AKT then triggers mTOR, a serine/threonine protein kinase existing in two distinct complexes: mTOR Complex 1 (mTORC1) and mTOR Complex 2 (mTORC2).^[^ ^103^, ^104^, ^105^, ^106^ ^]^ AKT activates mTORC1 by inhibiting the tumor suppressor TSC1/2, promoting key anabolic processes essential for cancer cell proliferation.^[^ ^107^ ^]^

The MAPK pathway, particularly the RAS/RAF/MEK/ERK cascade, represents another fundamental signaling axis transmitting extracellular signals from the cell surface to the nucleus, regulating cell division, differentiation, survival, and migration.^[^ ^108^, ^109^, ^110^ ^]^ This cascade is initiated by ligand binding to a cell surface receptor, leading to the sequential activation of RAS, followed by RAF, MEK, and finally ERK. Activated ERK then phosphorylates various transcription factors critical for gene expression. While mutations in core RAS/RAF/MEK/ERK genes are generally rare across all breast cancer subtypes, advanced molecular analyses have revealed significant non‐genetic activation mechanisms.^[^ ^111^, ^112^, ^113^ ^]^ In TNBC, specific mutations in Kirsten rat sarcoma viral oncogene homolog (KRAS) and B‐Raf proto‐oncogene, serine/threonine kinase (BRAF) are relatively higher, potentially compensating for the absence of growth signals from hormone receptors and Human Epidermal Growth Factor Receptor 2 (HER2).^[^ ^114^ ^]^ Genomic alterations dysregulating the MAPK pathway, including mutations in Mitogen‐Activated Protein Kinase 1 (MAP3K1) and Mitogen‐Activated Protein Kinase 4, amplifications of KRAS, BRAF, and Raf‐1 Proto‐Oncogene, Serine/Threonine Kinase, and truncations of Neurofibromin 1, have been strongly linked to immune‐silent (ICR Low) phenotypes, poor patient outcomes, and intrinsic treatment resistance.^[^ ^115^ ^]^ The increased expression of downstream molecules in the ERK and JNK cascades within immune‐silent tumors further highlights the activation of this pathway in driving aggressive breast cancer phenotypes.

### 2.3 The Role of the Tumor Microenvironment in Breast Cancer Progression

The TME is not merely a passive backdrop but a complex, dynamic ecosystem comprising diverse cellular and non‐cellular components that profoundly influence all stages of breast cancer, from initiation and progression to metastasis and treatment response.^[^ ^116^ ^]^ The TME actively shapes tumor behavior, serving as a critical determinant of disease prognosis and an increasingly important target for novel therapeutic interventions.^[^ ^117^ ^]^

Immune cells within the TME exert widespread effects on tumor progression and treatment outcomes. While cytotoxic T lymphocytes and M1 macrophages may initially offer growth‐inhibitory signals, breast cancer cells exhibit a remarkable capacity to “educate” and reprogram host stromal cells, including immune cells, toward pro‐tumorigenic characteristics.^[^ ^118^, ^119^, ^120^ ^]^ During tumor expansion, the TME orchestrates immune escape through multiple mechanisms. Activated cytokines stimulate the recruitment and differentiation of immune‐suppressive cells, such as regulatory T cells (Tregs) and myeloid‐derived suppressor cells (MDSCs).^[^ ^121^ ^]^ These cells actively suppress the function of anti‐tumor immune cells, thereby disrupting effective immune surveillance.^[^ ^122^ ^]^ Additionally, breast cancer cells can directly evade immune detection by overexpressing immune checkpoint ligands, notably Programmed Death‐Ligand 1 (PD‐L1).^[^ ^123^ ^]^ Cancer‐associated fibroblasts (CAFs) are among the most prominent and influential stromal cells in the TME, playing an indispensable and multifaceted role as architects and coordinators of tumor progression. CAFs secrete various signaling molecules and actively remodel the extracellular matrix (ECM) by depositing components and secreting ECM‐degrading enzymes.^[^ ^124^ ^]^ This remodeled ECM significantly promotes tumor growth and facilitates metastasis. The activation of the Yes‐associated protein (YAP)/Transcriptional Coactivator with PDZ‐binding Motif (TAZ) pathway in CAFs further contributes to matrix stiffening, influencing tumor behavior.^[^ ^125^ ^]^ CAFs also profoundly impact cancer progression by modulating the immune system, recruiting macrophages, and promoting their differentiation into the pro‐tumorigenic M2 phenotype, which is involved in phagocytosis, immune suppression, angiogenesis, and further ECM alterations.^[^ ^126^ ^]^ For instance, blocking the secretion of monocyte chemotactic protein 1 (MCP1) by CAFs can reduce monocyte recruitment to breast tumors.

The ECM is a complex and dynamic network providing structural support and regulating cellular behavior, composed of various components including collagen, fibrin, and proteoglycans.^[^ ^127^ ^]^ Within the TME, the ECM is a crucial non‐cellular component that significantly influences immune cell recruitment, survival, and function, thereby playing a key role in tumor progression and metastasis. Cancer cells acquire invasive characteristics through epithelial‐mesenchymal transition (EMT), enabling them to degrade the ECM via metalloproteinases and ultimately invade distant organs. The dense, collagen‐rich ECM within tumors, along with elevated interstitial fluid pressure (IFP), forms a notable physical barrier that significantly impedes the diffusion and penetration of nanocarriers to deeper tumor regions.^[^ ^128^, ^129^ ^]^ In turn, immune cells can directly or indirectly influence ECM remodeling by producing cytokines and chemokines; for example, macrophages contribute to ECM degradation via collagen uptake mediated by CD206 receptors.^[^ ^130^ ^]^

Ultimately, the TME is characterized by dynamic and bidirectional interactions between breast cancer cells and stromal cells. These interactions induce signaling pathways that promote tumor growth and survival through direct cell‐to‐cell contact or the release of autocrine and paracrine molecules.^[^ ^131^ ^]^ Moreover, future metastatic organs are not passive recipients of circulating tumor cells; rather, they are selectively and actively remodeled by the primary tumor prior to metastasis through the orchestration of a “pre‐metastatic niche” conducive to tumor cell survival and proliferation.^[^ ^132^, ^133^, ^134^, ^135^ ^]^ The inherent complexity of the TME and its central role in breast cancer progression make it a critical target for therapeutic intervention. A deep understanding of the TME, including the intricate crosstalk between its cellular and non‐cellular components, is therefore essential for developing novel strategies that can overcome resistance, enhance treatment efficacy, and effectively inhibit metastasis.

## 3 Current Therapeutic Strategies and Clinical Limitations in Breast Cancer

**Figure 4:**
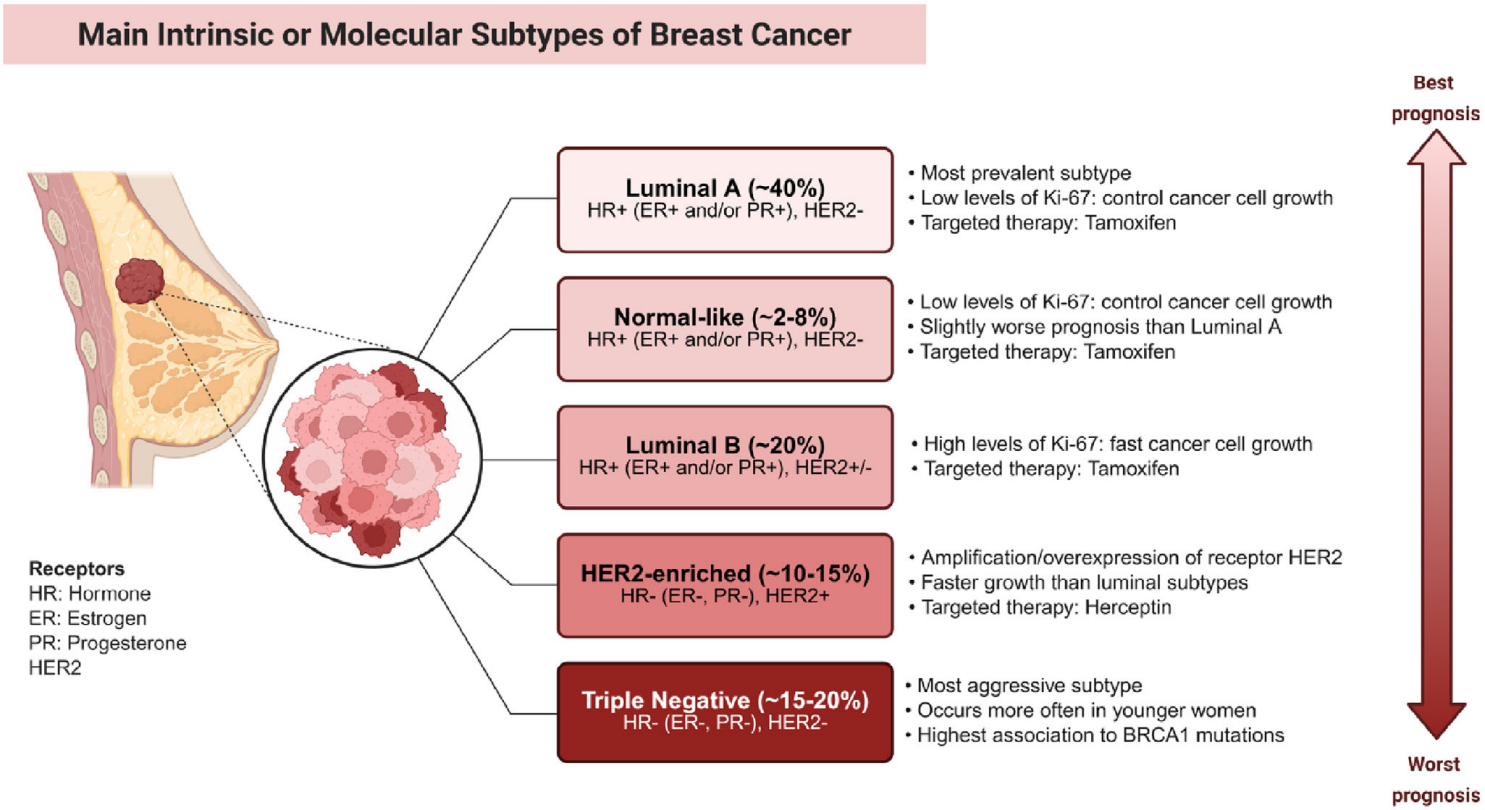
Schematic diagram of the main molecular subtypes of breast cancer.

**Figure 5:**
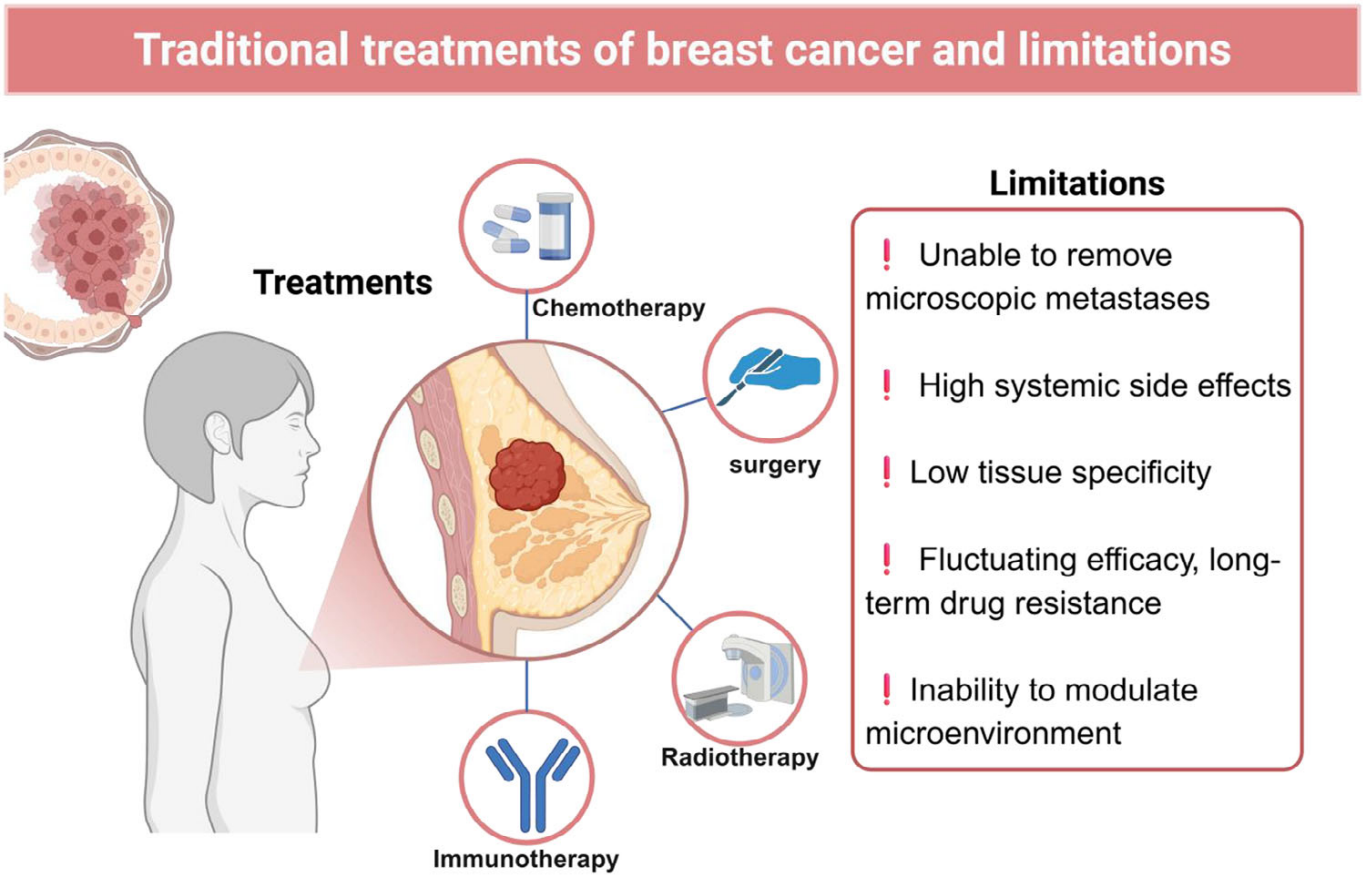
Schematic diagram of traditional treatment for breast cancer and its limitations.

Breast cancer can be categorized into distinct molecular subtypes with different biological behaviors (**Figure** **4**). Clinically, hormone receptor–positive (HR⁺) tumors, including luminal A and luminal B, generally show favorable prognosis and are responsive to endocrine therapy, although luminal B often exhibits higher proliferation and partial resistance. HER2‐positive tumors are characterized by aggressive growth but can be effectively treated with HER2‐targeted therapies such as trastuzumab. In contrast, triple‐negative breast cancer (TNBC) lacks hormone receptors and HER2 expression, shows the highest heterogeneity and metastatic potential, and has limited targeted treatment options, making it the most challenging subtype. The clinical management of breast cancer is a complex and multidisciplinary process, with the core focus on combining local and systemic treatments to maximize tumor clearance, prolong patient survival, and improve quality of life (**Figure** **5**). Local treatment primarily targets the removal of the primary tumor and its adjacent lymph nodes, while systemic treatment aims to systematically control potential micrometastases or distant metastases.^[^ ^136^, ^137^, ^138^ ^]^ The traditional strategies for treating TBCN and their advantages and disadvantages are summarized in **Table** **1**.

**Table 1:** Comparison Of Traditional Breast Cancer Therapies.

Treatment Modality	Advantages	Limitations
Surgery	Effective for localized tumors; enables complete tumor resection; can be curative in early stages	Invasive; may lead to complications; limited to local disease control
Radiotherapy	Reduces local recurrence; non‐invasive; improved precision with modern techniques	Associated with acute and chronic toxicity; less effective in hypoxic tumors
Chemotherapy	Systemic treatment; effective for aggressive and metastatic subtypes; often used as neoadjuvant/adjuvant	Significant side effects; resistance can develop; damage healthy cells
Endocrine Therapy	Highly effective for HR+ tumors; low systemic toxicity; can be used long‐term to prevent recurrence	Ineffective in HR‐ tumors; endocrine resistance is common; delayed onset of action
Targeted Therapy	Molecularly guided; effective for HER2+ and BRCA‐mutated tumors; fewer off‐target effects	Limited to patients with specific mutations; resistance and tumor escape mechanisms can occur; expensive

### 3.1 Surgical Approaches in Breast Cancer Management

Surgery remains the cornerstone of local treatment for breast cancer, aiming for complete resection of the primary tumor and comprehensive management of regional lymph nodes.^[^ ^139^, ^140^, ^141^ ^]^ Depending on tumor characteristics, patient preferences, and pathological diagnosis, the primary surgical approaches are breast‐conserving surgery (BCS) and mastectomy.^[^ ^142^, ^143^, ^144^ ^]^ Breast‐conserving surgery (BCS) aims to preserve breast shape and function while maintaining oncological outcomes comparable to mastectomy. It is typically combined with postoperative radiotherapy and is best suited for early‐stage, small, unifocal tumors with clear margins and limited ductal carcinoma in situ.^[^ ^145^ ^]^ Advances include oncoplastic techniques to improve aesthetics and sentinel lymph node biopsy (SLNB), which reduces lymphedema compared to axillary dissection and improves quality of life.^[^ ^146^ ^]^ Mastectomy, involving complete breast removal, remains essential for large or multicentric tumors, inflammatory cases, positive margins, high‐risk genetic carriers, recurrent disease, or patient preference. Modern approaches such as nipple‐ or skin‐sparing mastectomy allow immediate reconstruction and improve body image.^[^ ^147^, ^148^, ^149^ ^]^ Despite its foundational role, surgical treatment has inherent limitations. As an invasive procedure, it can lead to complications such as pain, infection, lymphedema, and breast deformity, impacting patients' quality of life.^[^ ^150^ ^]^ Crucially, surgery primarily addresses local disease and is ineffective against existing or potential distant micrometastases, necessitating subsequent systemic therapies. Furthermore, microscopic residual cancer cells at surgical margins can increase the risk of local recurrence. Beyond physical aspects, breast removal or alteration often imposes a significant psychological burden on patients due to its impact on body image.

### 3.2 Advances and Challenges in Breast Cancer Radiotherapy

Radiation therapy utilizes high‐energy ionizing radiation to eliminate cancer cells or inhibit their proliferation and remains a fundamental component of comprehensive breast cancer treatment.^[^ ^151^, ^152^, ^153^ ^]^ It plays a central role in multiple clinical settings, including breast‐conserving surgery, the management of locally advanced breast cancer, and palliation of metastatic disease.^[^ ^154^, ^155^, ^156^ ^]^ Radiation therapy has advanced significantly in recent years. Techniques such as 3D conformal radiotherapy (3D‐CRT) and intensity‐modulated radiotherapy (IMRT) improve dose precision and spare critical organs like the heart and lungs.^[^ ^157^, ^158^ ^]^ Image‐guided radiotherapy (IGRT) allows real‐time monitoring, while stereotactic body radiotherapy (SBRT) achieves high tumor control with limited toxicity. Proton and heavy ion therapy further reduce cardiac exposure, especially in left‐sided breast cancer.^[^ ^159^ ^]^ Radiation therapy strategies are increasingly tailored to the clinical stage and risk profile of patients. After breast‐conserving surgery, whole‐breast irradiation is the standard of care to reduce the risk of local recurrence.^[^ ^160^, ^161^, ^162^, ^163^, ^164^ ^]^ In metastatic disease, palliative radiotherapy relieves pain and prevents complications, improving quality of life.^[^ ^165^ ^]^ Nevertheless, radiotherapy has inherent limitations. Adverse effects may include acute toxicities such as skin dermatitis, esophagitis, and pneumonitis, as well as long‐term complications such as skin fibrosis, lymphedema, and cardiopulmonary dysfunction. Moreover, some tumors exhibit intrinsic resistance to radiation or may develop resistance during treatment. Tumor hypoxia is one of the primary mechanisms contributing to radioresistance.^[^ ^166^ ^]^ Recent studies have explored approaches to overcome this challenge by improving tumor oxygenation. For instance, platinum‐based nanozymes have been shown to enhance the efficacy of photodynamic therapy (PDT) by generating oxygen within the TME, offering a potential strategy to improve radiation response in hypoxic tumors. In addition, as a localized treatment, radiotherapy cannot eliminate distant micrometastases, emphasizing the importance of combining it with systemic therapies such as chemotherapy, endocrine therapy, or targeted agents. Finally, although the incidence is low, long‐term studies have reported a small risk of radiation‐induced secondary primary malignancies, underscoring the need for careful risk‐benefit assessment and long‐term monitoring.

### 3.3 Chemotherapeutic Regimens and Drug Resistance

Chemotherapeutic regimens for the treatment of TNBC primarily consist of anthracyclines, platinum‐based agents, and taxanes. Among these, anthracyclines such as doxorubicin (DOX) and epirubicin (EP) have demonstrated significant efficacy, improving treatment response rates and extending median survival by up to 22 months.^[^ ^167^, ^168^, ^169^ ^]^ Clinical trials confirmed their benefit in adjuvant and neoadjuvant settings, though long‐term efficacy is limited by recurrence and severe toxicities, including cardiotoxicity and bone marrow suppression.^[^ ^170^ ^]^ Although these regimens yielded high initial response rates, the long‐term benefits were limited by high recurrence and low overall survival rates. In addition, the use of anthracyclines is often constrained by acute toxicities, including irreversible cardiotoxicity, bone marrow suppression, alopecia, nausea, and vomiting, which impact treatment adherence and patient quality of life. Platinum‐based agents, either as monotherapy or in combination regimens, have also demonstrated promising outcomes in TNBC patients, particularly those carrying BRCA1 or BRCA2 mutations. A Phase II clinical trial (NCT0148694) evaluating single‐agent cisplatin showed a 21 percent pCR rate. When BRCA1 mutation carriers were excluded, the response rate decreased to 15 percent, underscoring the relevance of BRCA status in predicting therapeutic response. The enhanced sensitivity of BRCA‐mutated tumors to platinum agents is attributed to their impaired homologous recombination repair pathways.^[^ ^171^ ^]^ However, resistance can emerge, and combination strategies may be required to sustain therapeutic efficacy. Additional Phase II trials have supported the efficacy of combination therapies.^[^ ^172^, ^173^, ^174^ ^]^ Taxanes, alone or in anthracycline‐based combinations, achieve up to 40% complete pathological remission and help overcome drug resistance, solidifying their role in TNBC chemotherapy.

### 3.4 Emerging Role of Immunotherapy in Breast Cancer

Immunotherapy, which activates the host immune system to recognize and eliminate malignant cells, has emerged as a promising and innovative approach in cancer treatment.^[^ ^175^, ^176^, ^177^ ^]^ Multiple immunotherapeutic strategies have been developed, including immune checkpoint blockade (ICB), immunomodulators, cancer vaccines, and chimeric antigen receptor T‐cell (CAR‐T) therapy. These modalities have demonstrated notable clinical antitumor activity across various malignancies. Immunomodulators, such as cytokines, interleukins, chemokines, and immunoregulatory drugs, can modulate the immune microenvironment and enhance the functional activity of immune cells. Cancer vaccines, which typically incorporate tumor‐associated antigens and immunostimulatory adjuvants, are designed to elicit tumor‐specific immune responses.^[^ ^178^ ^]^ In addition to promoting immediate tumor clearance, these vaccines may induce long‐term immune memory, potentially reducing recurrence rates. Numerous therapeutic cancer vaccines are currently under clinical investigation. CAR‐T cell therapy, which involves the genetic modification of T cells to express chimeric receptors that recognize tumor‐specific antigens, has shown remarkable efficacy in hematologic malignancies.^[^ ^179^, ^180^, ^181^ ^]^ These receptors integrate antigen‐binding domains with intracellular signaling and co‐stimulatory motifs, enabling robust T‐cell activation and cytotoxicity. Among immunotherapeutic strategies, immune checkpoint blockade has received significant attention for its ability to reinvigorate exhausted T cells. This is achieved by targeting inhibitory pathways, most notably those mediated by cytotoxic T‐lymphocyte‐associated protein 4 (CTLA‐4) and programmed death‐1 (PD‐1). Checkpoint inhibitors targeting these pathways are now approved for the treatment of various cancers and are under extensive investigation for TNBC. The PD‐1/PD‐L1 axis has become a focal point in TNBC immunotherapy.^[^ ^182^ ^]^ PD‐1 is a receptor expressed on T cells that, upon binding to its ligand PD‐L1 on tumor cells, suppresses T‐cell function and facilitates immune evasion. PD‐1 inhibitors disrupt this interaction, thereby restoring antitumor immune activity.^[^ ^183^, ^184^, ^185^ ^]^ Among these inhibitors, pembrolizumab has demonstrated clinical benefit in patients with PD‐L1‐positive TNBC and is approved for use in combination with chemotherapy as a first‐line treatment. Clinical trials have provided evidence of pembrolizumab's efficacy. In one trial, patients with PD‐L1‐positive TNBC who received pembrolizumab in combination with neoadjuvant or adjuvant chemotherapy achieved a median overall survival of 16.1 months, with 87 percent of participants receiving concurrent taxane‐ or anthracycline‐based regimens. In a Phase II clinical study (NCT01042379), a cohort of 69 HER2‐positive and 29 TNBC patients received neoadjuvant paclitaxel with or without pembrolizumab, followed by dose‐adjusted doxorubicin and cyclophosphamide.^[^ ^186^ ^]^ The pathological complete response rate increased to 71 percent in the pembrolizumab‐treated group, compared to 19 percent in the control group. Another analysis of the same trial reported an estimated complete response rate of 62 percent in the pembrolizumab arm versus 22 percent in the control arm. Collectively, these findings underscore the therapeutic potential of immunotherapy in TNBC. However, challenges such as immune‐related adverse events, limited response rates in PD‐L1‐negative tumors, and mechanisms of resistance remain areas of active investigation. Ongoing research is focused on identifying predictive biomarkers, optimizing combination regimens, and expanding immunotherapy to broader patient populations.

### 3.5 Targeted Therapy

Lehmann et al. classified TNBC into six molecular subtypes based on comprehensive gene expression profiling: basal‐like 1, basal‐like 2 immunomodulatory, mesenchymal, mesenchymal stem‐like, and luminal androgen receptor subtypes. This molecular heterogeneity underscores the need for subtype‐specific targeted therapies in TNBC.^[^ ^187^, ^188^ ^]^ In recent years, significant efforts have been directed toward developing targeted therapeutic strategies for TNBC, particularly focusing on key oncogenic pathways such as poly(ADP‐ribose) polymerase (PARP), phosphoinositide 3‐kinase/protein kinase B (PI3K/AKT), mitogen‐activated protein kinase (MAPK), and cyclin‐dependent kinase (CDK) pathways. Among these, PARP inhibitors have shown remarkable promise, especially in TNBC patients harboring BRCA1 or BRCA2 mutations. PARP‐1 inhibitors exacerbate DNA double‐strand breaks by impairing single‐strand DNA repair, ultimately promoting apoptosis in tumor cells.^[^ ^189^, ^190^, ^191^ ^]^ Although BRCA mutations are present in only ≈20% of TNBC patients, several PARP inhibitors have received regulatory approval due to their clinical efficacy. Olaparib was the first FDA‐approved PARP inhibitor for BRCA‐mutated advanced TNBC, following a Phase III clinical trial that demonstrated an improvement in progression‐free survival (PFS) to 7.0 months compared to 4.2 months with placebo. Talazoparib, another potent PARP inhibitor, was approved based on a Phase I trial (NCT03499353), which reported a median overall survival (OS) of 24.3 months in HER2‐negative advanced breast cancer patients, significantly outperforming the 6.3 months observed in those receiving standard chemotherapy. The PI3K/AKT signaling pathway, which governs critical cellular processes such as growth, survival, and glucose metabolism, is frequently dysregulated in TNBC.^[^ ^192^, ^193^, ^194^ ^]^ Despite its high activation frequency, the pathway exhibits complex regulatory dynamics. Ipatasertib, a selective oral AKT inhibitor, has shown encouraging results in clinical studies. In the Phase II LOTUS trial (NCT02162719), the combination of ipatasertib with paclitaxel extended PFS from 4.9 months to 9.0 months and improved OS from 18.4 to 23.1 months in patients with advanced TNBC. Additionally, inhibition of the epidermal growth factor receptor (EGFR), a transmembrane tyrosine kinase that signals through the PI3K/AKT cascade, has also demonstrated therapeutic potential in EGFR‐overexpressing TNBC cases. Collectively, these targeted approaches reflect a growing shift toward precision oncology in TNBC, emphasizing the need to match molecular subtypes with pathway‐specific inhibitors to improve clinical outcomes.

## 4 Nanozyme‐Based Therapeutic Strategies for Breast Cancer

Nanozymes, due to their unique catalytic activity and controllability, show tremendous potential in breast cancer treatment. They can be designed to overcome the limitations of traditional therapies and enhance synergy with various therapeutic modalities.^[^ ^195^, ^196^, ^197^, ^198^, ^199^ ^]^ This section reviews recent advances in nanozyme‐based breast cancer therapies, highlighting their applications in immunotherapy, chemotherapy, phototherapy, and combination strategies, as well as discussing the underlying mechanisms that contribute to their efficacy. Some characteristics and advantages of nanozymes for treating TBCN are summarized in **Table** **2**.

**Table 2:** Characteristics and advantages of different representative nanozymes.

Nanozyme Material	Typical Enzyme‐Mimetic Activity	Catalytic/Functional Features
Mn	Catalase, Peroxidase, Glucose oxidase	TME‐responsive, ROS amplifier, hypoxia modulation
Fe	Peroxidase, Fenton‐like, Oxidase	Strong ROS generation, suitable for ICD induction
Ce	Catalase, Oxidase, ROS scavenger	High redox cycling, anti‐inflammatory, low toxicity
Pt	Catalase, SOD‐like, Oxygen modulation	Efficient O_2_ generation, dual‐mode imaging capability
Au	Peroxidase, Laccase‐like, PTT enhancer	High photothermal conversion, biocompatible
Cu	Peroxidase, Fenton‐like, Glucose depletion	Fenton reaction initiator, effective in nutrient depletion
Boron	Peroxidase, H_2_O_2_ responsive, Non‐metal catalytic	Biodegradable, metal‐free, safe for long‐term use
Carbon	Peroxidase, Oxidase, Electron relay	Surface‐tunable, carrier‐friendly, high electron mobility
Co	Peroxidase, Superoxide dismutase (SOD)‐like	SOD mimicry, enhances oxidative stress selectively
V	Haloperoxidase‐like, Oxidase, ROS regulator	Mimics haloperoxidase for antimicrobial/antitumor effect
Ni	Peroxidase, Electrocatalytic ROS generation	Redox catalysis under mild conditions, PTT compatible
MOFs	Multienzyme‐mimic (POD, CAT, Oxidase)	Highly porous, excellent loading and co‐delivery potential
Prussian Blue	Catalase, Peroxidase, Inflammation modulator	Clinically used, anti‐inflammatory, FDA‐approved base
Polyoxometalates	Multifunctional redox enzymes (POD, Oxidase, Reductase)	High valency clusters, versatile redox chemistry
Ti	Photocatalytic oxidase, Peroxidase under UV/visible light	Photoresponsive, ROS regulation under light control

### 4.1 Nanozymes for Enhancing Tumor Immunogenicity and Immune Activation

Tumor immunotherapy has made tremendous progress, but it still faces serious challenges, namely, it is only effective in a relatively small group of patients, and the low immunogenicity of tumors also limits the effectiveness of immunotherapy. The efficacy of immunotherapy is closely related to tumor immunogenicity. Tumors with high immunogenicity are referred to as “hot” tumors, typically characterized by an immunogenic microenvironment, including T‐cell infiltration, cytokine production, and high PD‐L1 expression.^[^ ^200^ ^]^ In contrast, tumors with low immunogenicity are termed “cold” tumors, typically associated with a non‐immunogenic microenvironment.^[^ ^201^ ^]^ Research has confirmed that only “hot” tumors respond well to immunotherapy, while “cold” tumors can evade immune attacks. Currently, there are no standard methods to accurately quantify the degree of tumor immunogenicity, but tumor immunogenicity can be qualitatively analyzed using representative T cells and cytokines, and sometimes clinical outcomes can also be used as a reference for judgment. TNBC, pancreatic cancer, and prostate cancer have all been confirmed to be “cold” tumors with low immunogenicity. Therefore, enhancing tumor immunogenicity to improve the recognition and expression of tumor‐associated antigens is the key to activating the anti‐tumor immune response.

**Figure 6:**
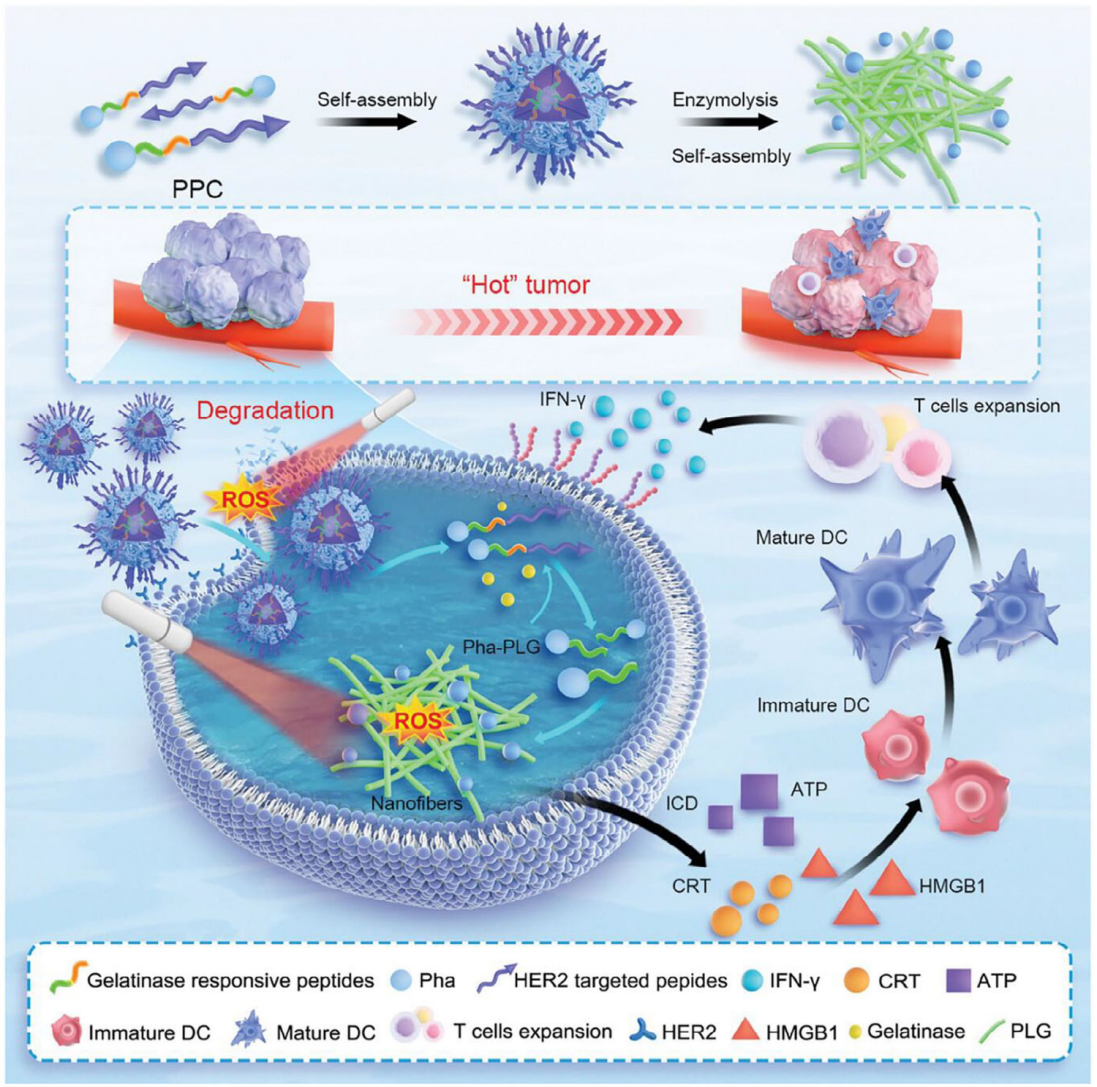
Schematic diagram of self‐assembled PPC nanoparticles actively recognizing and targeting breast cancer cells through HER2‐targeted peptides, thereby enhancing immunotherapy. Reproduced with permission.^[^ ^202^ ^]^ Copyright 2024, Wiley‐VCH.

Guo et al.^[^ ^202^ ^]^ provide an innovative case study. They introduce an innovative strategy for enhancing breast cancer immunotherapy by precisely degrading HER2 protein through peptide‐conjugated PDT, which can be used as a case study for nanozyme‐mediated breast cancer treatment. At the core of this strategy is a self‐assembled nanoparticle (PPC) that enhances the immune response through the following mechanisms. HER2 protein degradation and induction of immunogenic cell death (ICD): PPC nanoparticles actively recognize and target breast cancer cells via HER2‐targeted peptides. Under near‐infrared (NIR) light irradiation, the photosensitizer (Pha) within the nanoparticles generates reactive oxygen species (ROS), effectively degrading the HER2 protein on the surface of cancer cells. This PDT not only directly kills tumor cells but also induces ICD. The occurrence of ICD releases key damage‐associated molecular patterns (DAMPs), such as adenosine triphosphate (ATP), high‐mobility group box 1 (HMGB1), and calcium‐releasing transmembrane protein (CRT). These DAMPs act as “danger signals” that can be recognized by dendritic cells (DCs), thereby promoting antigen presentation and the activation of immune cells. Activation of DCs and T cell responses: The release of ICD signals promotes the maturation of immature dendritic cells and stimulates them to secrete immune‐activating cytokines, such as tumor necrosis factor‐α (TNF‐α) and interleukin‐6 (IL‐6). These cytokines are crucial for dendritic cell maturation, antigen presentation, and T cell activation. Enhancing cytotoxic T cell (CD8^+^ T cell) activity: As DCs become activated and their antigen presentation capacity increases, the immune environment changes, leading to a reduction in the number of regulatory T cells (Foxp^3+^/CD4^+^) and an increase in the number of cytotoxic T cells (CD8^+^ T cells). CD8^+^ T cells subsequently secrete interferon‐γ (IFN‐γ), further enhancing the activity of immune cells and driving a robust antitumor immune response. In vitro experiments demonstrated that, compared to standalone PDT, PPC nanoparticles at nanomolar (nM) concentrations significantly enhance the degradation efficiency of HER2 protein. In vivo experiments confirmed that PPC nanoparticles exhibit excellent biosafety, tumor targeting, and efficient HER2 degradation capabilities. Notably, in terms of immune enhancement, tumor mouse models treated with PPC nanoparticles and phototherapy showed approximately a threefold increase in the number of tumor‐infiltrating CD8^+^ T cells compared to the control group, while the number of inhibitory regulatory T cells (Tregs) was significantly reduced. This enhanced cytotoxic T cell activity effectively inhibited tumor growth, and a complete tumor regression rate of more than 50% was observed in some models, significantly improving the anti‐tumor immune response and therapeutic effect. After PPC nanoparticles are cleaved by gelatinase in tumor cells, the released Pha‐PLG molecules self‐assemble to form nanofibers. These nanofibers have a larger diameter and are not easily expelled by cells, thereby prolonging their circulation time in the body. The extended circulation time facilitates sustained immune induction and activation of CD8^+^ T cells, thereby establishing a robust tumor‐immune feedback loop. In summary, this peptide‐conjugated PDT‐based nanoparticle significantly enhances antitumor immune responses by targeting HER2 protein degradation, inducing ICD, activating dendritic cells, and enhancing cytotoxic T cell activity, thereby providing a new therapeutic approach for breast cancer treatment. (**Figure** **6**)

**Figure 7:**
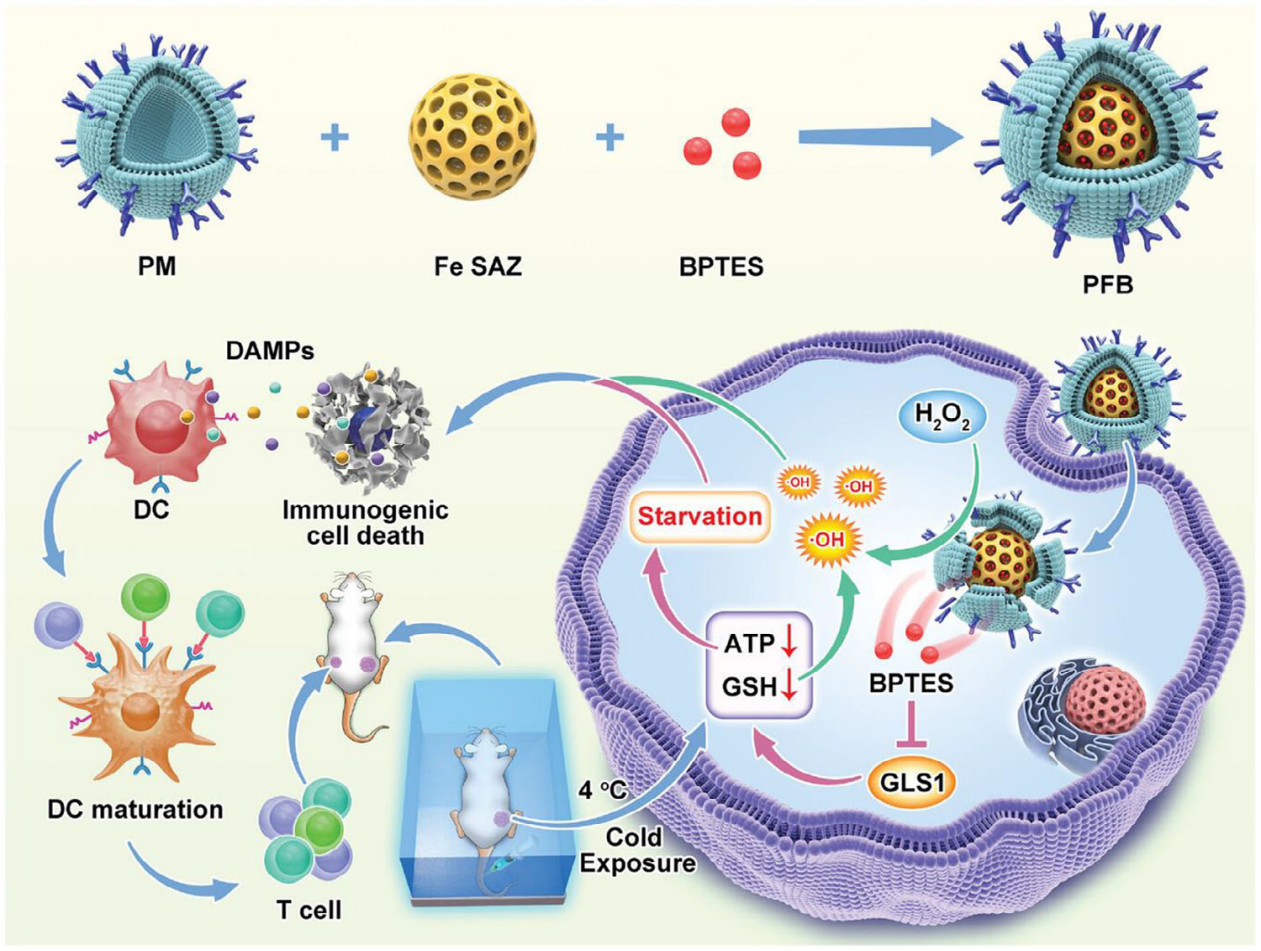
Schematic diagram of biomimetic single‐atom nanozymes used in dual‐hunger‐enhanced breast cancer immunotherapy. Reproduced with permission.^[^ ^203^ ^]^ Copyright 2024, Wiley‐VCH.

In another study, Zhan et al.^[^ ^203^ ^]^ explored the application of a biomimetic single‐atom nanozyme (SAE) in enhancing breast cancer immunotherapy (**Figure** **7**). The core design concept was to construct a platelet membrane‐mimetic nanomedicine (PFB) and combine it with cold exposure (CE) therapy to achieve a dual starvation strategy targeting tumors. The PFB nanomedicine is loaded with the glutaminase inhibitor BPTES and is designed to achieve precise targeting of tumors through the biomimetic properties of platelet membranes. After entering cancer cells, the Fe‐SAEs in PFB exhibit POD activity, which can catalyze H2O2 to generate hydroxyl radicals (•OH), thereby increasing the level of ROS in cells. Concurrently, the synergistic effect of CE therapy and BPTES induces a significant reduction in glucose and ATP levels in cancer cells, leading to extensive depletion of intracellular glutathione, thereby enhancing the cytotoxic effects of ROS and placing tumor cells in a “starvation” state, making them more sensitive to oxidative stress. In terms of quantitative results, this study achieved significant progress: in vitro experiments confirmed that compared to SAEs therapy alone, the combination of PFB and CE more effectively induces ICD, manifested by a significant increase in CRT exposure and high‐mobility group box 1 (HMGB1) release. In an in vivo mouse model, this combination therapy significantly promoted the maturation of DCs in lymph nodes, activated 75% of DC cells, and increased the number of cytotoxic CD8^+^ T cells by ≈2.5‐fold, while also significantly enhancing the abundance of memory T cells. Ultimately, this robust immune activation not only reduced the volume of primary tumors by an average of over 70% but also significantly inhibited the growth of distant tumors and tumor metastasis, with the number of metastatic lesions decreasing by an average of 80% compared to the control group. This study offers an innovative paradigm for multimodal synergistic therapy. By combining biomimetic nanotechnology, metabolic starvation strategies, and nanozyme catalytic activity, it successfully killed tumor cells while effectively activating the body's anti‐tumor immune response. This provides an important theoretical basis and experimental evidence for the future design and development of next‐generation nanomedicines to overcome the limitations of traditional tumor treatments, especially by regulating the TME and enhancing the immune response to achieve more efficient and longer‐lasting cancer treatments.

Overall, nanozymes show significant promise in breast cancer immunotherapy by serving as multifunctional platforms that integrate diverse treatment strategies with immune activation. This approach overcomes the limitations of single modalities and achieves synergistic “multi‐target” effects. For instance, PPC nanoparticles enhance tumor immunogenicity by degrading HER2 and inducing ICD through PDT, while PFB nanozymes remodel the microenvironment via catalytic activity and metabolic starvation. Nanozymes can also intelligently regulate the TME, responding to specific conditions for precise drug release and localized activation, thereby improving therapeutic specificity and reducing systemic toxicity. Importantly, these strategies emphasize inducing ICD, either through ROS from PDT or oxidative stress driven by nanozyme catalysis and metabolic disruption. Both mechanisms trigger DAMP release, transforming “cold” tumors into “hot” tumors and enhancing anti‐tumor immunity. Through DAMP release and co‐delivery of immunomodulators such as IFN‐γ, nanozymes promote dendritic cell maturation, boost antigen presentation, expand CD8⁺ T cell infiltration, and reduce immunosuppressive cells, reversing TME suppression. Moreover, size effects and surface modifications, including platelet membrane mimicry and HER2‐targeted peptides, enable selective tumor accumulation and localized drug delivery, maximizing efficacy while minimizing toxicity. Beyond controlling primary tumors, nanozyme‐based immunotherapy can establish systemic immune memory, effectively suppressing recurrence and metastasis. In summary, these findings highlight nanozymes as versatile and intelligent platforms with great potential to enhance immunogenicity, activate immune responses, and improve long‐term survival in breast cancer patients.

### 4.2 Nanozyme‐Driven Drug Delivery and Chemotherapy Sensitization

In the chemotherapy of breast cancer, although traditional chemotherapy drugs have strong cytotoxicity, their widespread systemic toxicity, tumor resistance, and poor targeting severely limit clinical efficacy and cause significant side effects for patients.^[^ ^204^ ^]^ The emergence of nanozymes provides an innovative way to overcome these limitations. Through clever design, nanozymes can act as smart carriers to accurately deliver chemotherapy drugs to tumor sites, achieving a high local concentration of drugs in tumors, thereby significantly improving treatment efficacy and reducing damage to healthy tissues. More importantly, the enzyme‐mimicking activity of nanozymes can synergize with chemotherapy drugs.^[^ ^205^ ^]^ For example, some nanozymes can regulate the TME, enhance the sensitivity of tumor cells to chemotherapy drugs, or directly induce cell death, thereby overcoming resistance mechanisms. In addition, nanozymes can also respond to TME‐specific stimuli to achieve on‐demand release of chemotherapy drugs, further improving the accuracy and safety of treatment.^[^ ^206^ ^]^ This strategy of combining the multifunctionality of nanozymes with traditional chemotherapy provides a more efficient, targeted, and less side‐effect treatment option for breast cancer, which is expected to significantly improve patient prognosis.

**Figure 8:**
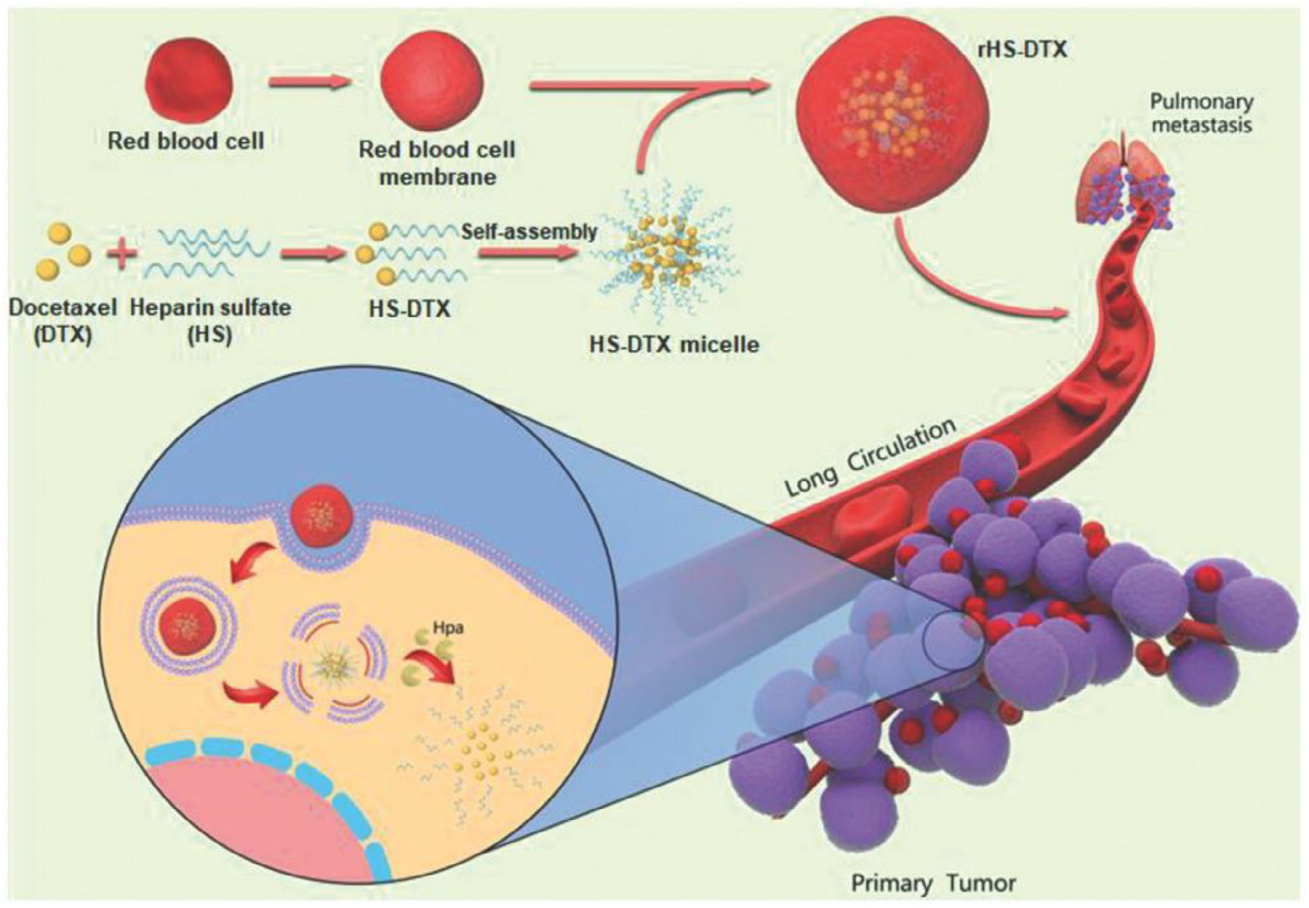
Schematic diagram of the synthesis of rHS‐DTX and the mechanism of enhancing the anticancer efficacy of DTX and inhibiting metastasis. Reproduced with permission.^[^ ^207^ ^]^ Copyright 2020, Wiley‐VCH.

Lang et al.^[^ ^207^ ^]^ designed a tumor cell‐selective bionic nanodevice (rHS‐DTX) to enhance the anticancer efficacy of the chemotherapy drug docetaxel (DTX) and inhibit metastasis by utilizing heparinase (Hpa), which is overexpressed in breast cancer cells. The core design concept of this nanodevice is to overcome the limitations of traditional chemotherapy drugs, which have poor selectivity for cancer cells, significant side effects, and difficulty in effectively accumulating in tumor sites. Researchers constructed a biomimetic nanodevice (rHS‐DTX) composed of docetaxel‐heparin sulfate (DTX‐HS) conjugate micelles coated with red blood cell (RBC) membranes. DTX is covalently linked to heparin sulfate (HS), forming the HS‐DTX prodrug. In normal cells, HS masks the cytotoxicity of DTX, keeping its activity in a “hidden” state. However, in cancer cells that highly express heparinase (Hpa), Hpa can specifically hydrolyze HS, thereby “restoring” and releasing active DTX within the tumor. This enzyme‐responsive release mechanism ensures tumor‐specific activation of the drug and is key to nano‐enhanced chemotherapy. Coating the HS‐DTX micelles with RBC membranes confers longer circulation time and higher tumor cell uptake efficiency to the nanodevice. The “self‐marking” proteins on the RBC membrane help the nanoparticles evade clearance by the mononuclear phagocyte system and immune attacks. This biomimetic strategy significantly improves the pharmacokinetics and biodistribution of DTX in vivo, ensuring that more drug reaches the tumor site. In an MCF‐7 metastatic breast cancer mouse model, the intratumoral accumulation of DTX in the rHS‐DTX group was 6.35 times higher than that in the free DTX injection group. Eight hours post‐administration, the DTX content in tumors of the rHS‐DTX group was 6.35 times and 1.76 times higher than that in the free DTX and HS‐DTX groups, respectively. rHS‐DTX achieved a tumor inhibition rate of 98.2%. The nanodevice demonstrated a lung metastasis inhibition rate of 99.6%, nearly completely preventing the formation of lung metastasis lesions, and no severe toxicity was observed in major organs or blood in mice treated with rHS‐DTX. rHS‐DTX exhibited cytotoxicity comparable to free DTX in MCF‐7 cancer cells (IC50 value of 0.57 µg mL^−1^), but extremely low cytotoxicity in non‐cancerous MCF‐10A cells, demonstrating tumor cell selectivity. Within 48 h, 97.87% of HS‐DTX was converted by Hpa and released DTX. This work provides important inspiration for nano‐enhanced chemotherapy. Although the study did not synthesize inorganic nanozymes in the traditional sense, it cleverly utilized naturally occurring enzymes (Hpa) overexpressed in the TME as “biological nanozymes” to precisely catalyze the activation of prodrugs. This shows that, in addition to designing artificially synthesized nanozymes, using endogenous enzymes to drive the specific release and activation of drugs is also a very promising “nanozyme‐enhanced” strategy. By using enzymes specifically overexpressed in the TME as trigger factors, the study achieved intelligent, precise release and activation of chemotherapy drugs. This represents a more effective and safer drug delivery paradigm, significantly increasing drug concentration at the tumor site while avoiding damage to normal tissues. The use of biological materials such as red blood cell membranes endows the nanocarriers with excellent long‐circulation properties and tumor‐targeting capabilities. This biomimetic strategy significantly improves drug bioavailability and tumor accumulation, representing a key technological breakthrough in enhancing chemotherapy efficacy. The study not only focuses on inhibiting primary tumors but also emphasizes the prevention and treatment of metastatic breast cancer. By achieving high DTX accumulation in tumors and the lungs, the formation of metastatic lesions is effectively inhibited, providing a promising strategy for treating metastatic cancers. In summary, this study provides an efficient, accurate, and safe model for nano‐enhanced chemotherapy by cleverly utilizing natural enzymes in the TME as mediators for targeted activation, opening up new avenues for the future development of smart, responsive anti‐cancer nanomedicines. (**Figure** **8**)

**Figure 9:**
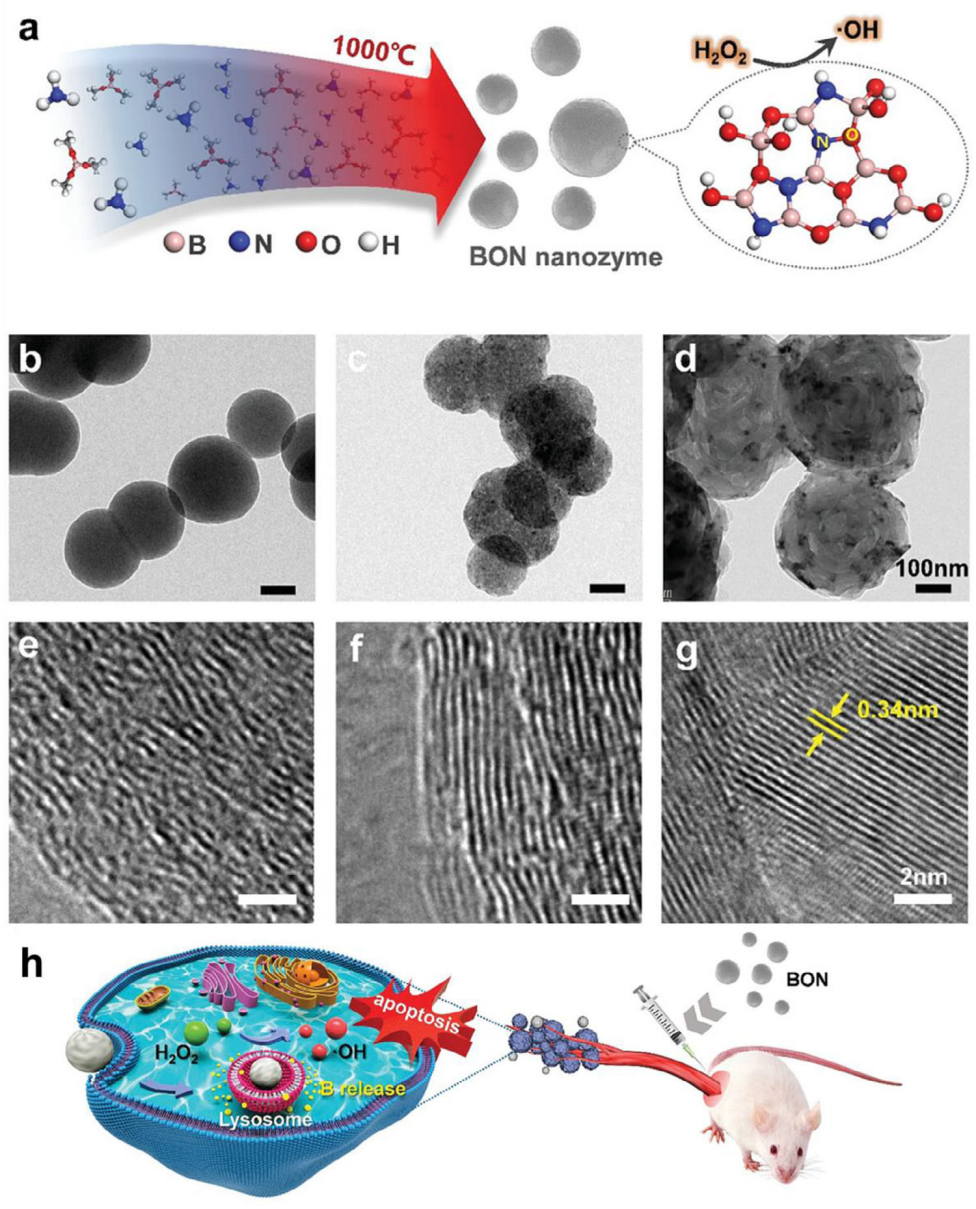
Schematic diagram of BON nanosphere‐enhanced immunotherapy for breast cancer. a) Schematic diagram of BON nanosphere pyrolysis synthesis. b–d) Transmission electron microscopy (TEM) images of BON, BON1000, and BON1400. Scale bar: 100 nm. e–g) High‐magnification TEM images of BON, BON1000, and BON1400. h) Schematic diagram of the therapeutic mechanism of BON. Scale bar: 2 nm. Reproduced with permission.^[^ ^208^ ^]^ Copyright 2021, Wiley‐VCH.

Zeng et al.^[^ ^208^ ^]^ developed a novel biodegradable peroxidase‐mimicking boron‐oxygen‐nitrogen (BON) nanozyme for efficient multimodal breast cancer treatment. The core design of this study is to use BON nanostructures as artificial enzymes to mimic the activity of natural peroxidases, thereby achieving self‐healing nanomedicines that do not require external stimuli or drugs. BON nanozymes can catalyze the conversion of high levels of H_2_O_2_ in tumor cells into highly cytotoxic hydroxyl radicals (·OH). Due to their rapid proliferation, tumor cells usually accumulate large amounts of hydrogen peroxide and are more sensitive to ROS than normal tissues because of their lower levels of antioxidant enzymes. This characteristic makes the generation of ROS through endogenous H_2_O_2_ a promising anti‐cancer strategy. BON nanozymes are designed to be biodegradable materials. Studies have shown that BON nanozymes can be almost completely degraded in aqueous solution within 7 days, producing boric acid and NH_4_B_6_O_8_·4H_2_O. This solves the toxicity problems that may be caused by the long‐term retention of nanomaterials in the body and enhances their potential for clinical translation. By catalyzing the generation of hydroxyl radicals, BON nanozymes can induce cancer cell apoptosis, thereby achieving the treatment of breast cancer. This endogenous H_2_O_2_‐based autocatalytic mechanism avoids the limitations of traditional PTT, PDT, or magnetic therapy, which require external stimulation, making it a non‐invasive and self‐healing nanomaterial. BON nanozyme has shown significant anti‐cancer effects in both in vitro and in vivo experiments. BON nanozyme can significantly reduce the viability of 4T1 cancer cells. Within 48 h, it reduces cancer cell viability by 82% through the induction of apoptosis. At a BON concentration of 50 µg mL^−1^, cell viability drops to 50%; when the concentration increases to 400 µg mL^−1^, cell viability further decreases to 18%. In contrast, inert or B‐releasing boron nitride (BN) nanospheres showed significantly lower cytotoxicity. In vivo experiments confirmed the high efficacy of BON nanozymes in inhibiting breast tumor growth. After 14 days of treatment, BON nanozymes achieved a 97% breast tumor growth inhibition rate compared to the control group. This effect was 10 times higher than that of inert BN nanospheres and 1.3 times higher than that of B‐releasing BN nanospheres. BON nanozymes have good biocompatibility. Mouse serum biochemical analysis and H&E staining experiments showed that BON nanozymes had no significant effect on mouse liver function after 7 days of treatment, indicating that they have good biocompatibility with normal tissues. This study provides important insights into the design of nanozymes and cancer treatment in many ways: It proposes a concept of “self‐healing” nanozymes that can exert therapeutic effects without drug loading or external stimulation. This simplifies the treatment process, reduces complexity, and avoids potential side effects and equipment requirements associated with external stimulation, facilitating clinical translation. It cleverly utilizes the high levels of H_2_O_2_ in tumor cells as a “substrate” and converts it into harmful ROS through the catalytic activity of nanozymes, achieving specific killing of tumors. This provides a new strategy for precision treatment based on the characteristics of the TME. It emphasizes the importance of the biodegradability of nanomaterials and addresses the safety hazards of long‐term accumulation in the body. This is crucial for the clinical application of nanomedicines, as it ensures that nanomaterials can be effectively cleared after completing their therapeutic tasks. This is the first report of boron oxynitride (BON) nanostructures as a new type of peroxidase‐mimicking nanozyme, and confirms their high efficiency in the treatment of breast cancer. This expands the scope and potential of boron‐based nanomaterials in biomedical applications, particularly in the field of cancer treatment. (**Figure** **9**)

### 4.3 Nanozyme‐Augmented Photothermal and Photodynamic Therapies

PTT is a non‐invasive, highly controllable, and precise treatment modality that leverages photothermal agents to convert absorbed photon energy into heat upon laser irradiation, thereby inducing thermal ablation of cancer cells when local temperatures exceed 42–45°C.^[^ ^209^, ^210^ ^]^ NIR light is preferred for its deeper tissue penetration and reduced absorption/scattering by biological tissues. While first near‐infrared (NIR‐I) lasers are commonly used, second near‐infrared (NIR‐II) lasers offer superior advantages due to even deeper penetration, higher spatial resolution, and lower biological tissue damage. PDT is another emerging minimally invasive approach gaining significant traction, particularly in oncology. It relies on three fundamental components: a photosensitizer (PS) localized within the tumor, a light source of an appropriate wavelength, and endogenous oxygen. Individually non‐toxic, their combined photochemical reaction generates highly cytotoxic ROS, including singlet oxygen, leading to tumor cell death via apoptosis or necrosis. Clinically, PDT is applied for precancerous lesions, skin cancer, and solid tumors such as esophageal, lung, prostate, and breast cancers. Beyond direct cytotoxicity, PDT is recognized for its ability to induce ICD. This process involves the exposure or release of DAMPs, which act as “danger signals.” These DAMPs can override tumor immune evasion mechanisms and activate the innate immune system, leading to the engagement of macrophages, dendritic cells, natural killer cells, and neutrophils.^[^ ^211^, ^212^, ^213^ ^]^ The subsequent activation of the innate immune system further stimulates adaptive immunity, promoting the recruitment and activation of antigen‐presenting cells, and enhancing the infiltration of helper T cells and cytotoxic T cells at the tumor site. Nanozymes significantly enhance both PTT and PDT, overcoming inherent limitations and boosting therapeutic efficacy. In PTT, nanozymes can function as efficient photothermal agents themselves, converting absorbed light into localized heat to ablate tumors.^[^ ^32^, ^214^, ^215^ ^]^ For instance, certain metal oxide nanozymes or those integrated into hydrogels can exhibit excellent photothermal conversion efficiencies. In PDT, nanozymes critically address tumor hypoxia, a major barrier to treatment effectiveness. By mimicking natural enzymes like catalase, some nanozymes can convert endogenous hydrogen peroxide within the hypoxic TME into oxygen, thereby alleviating hypoxia and enhancing ROS generation for the photosensitizer. Alternatively, peroxidase‐mimetic nanozymes can directly generate cytotoxic ROS, synergizing with or augmenting the effects of photosensitizers in PDT. This dual capability of providing heat and enhancing ROS production (PDT) allows nanozymes to serve as versatile platforms for highly effective combination therapies, enabling precise, multi‐modal cancer treatment strategies.

**Figure 10:**
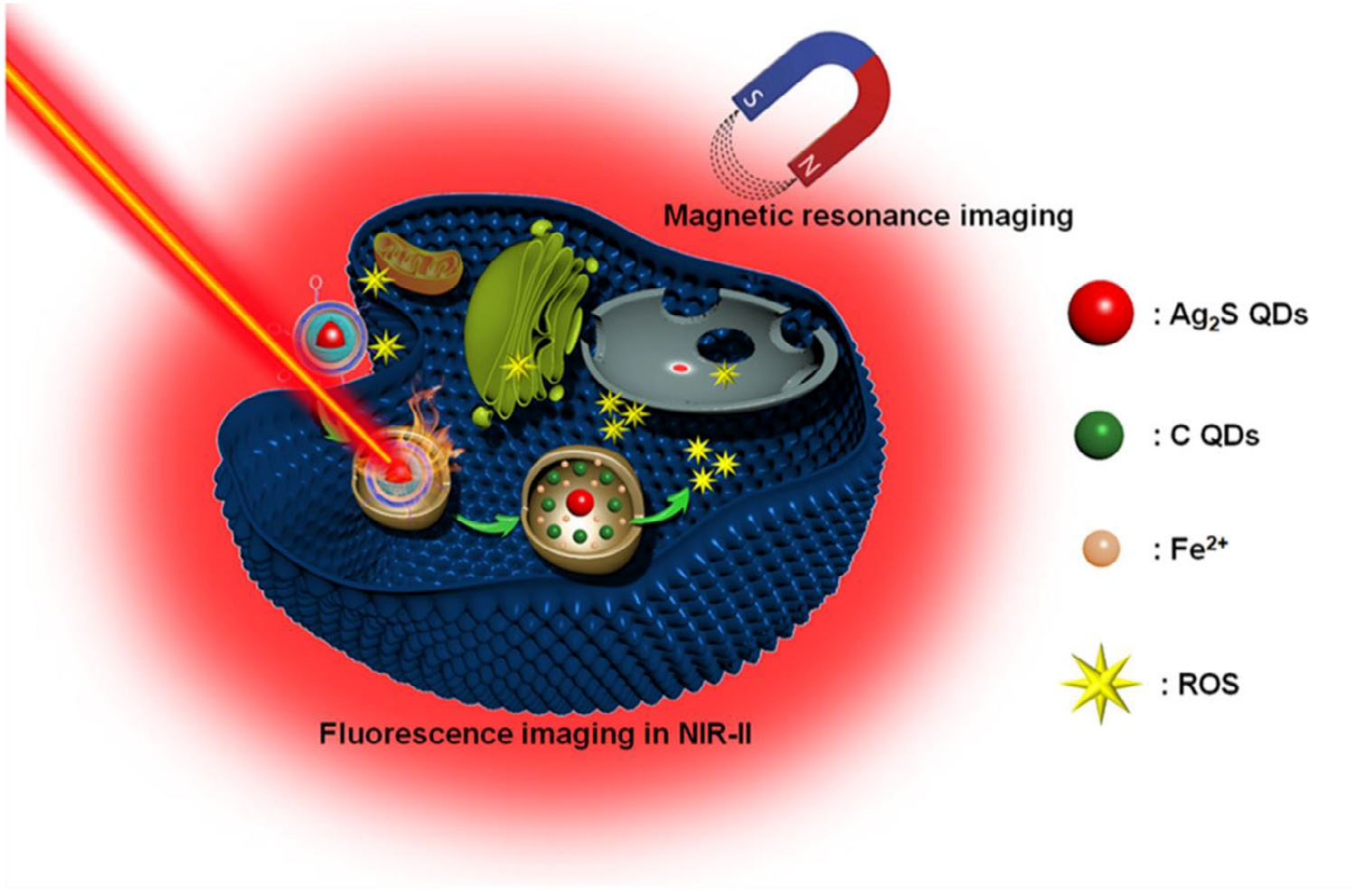
Schematic representation of the biochemical processes of multifunctional Ag2S@Fe2C‐DSPE‐PEG‐iRGD within breast cancer cells. PTT: Photothermal Therapy. Reproduced with permission.^[^ ^216^ ^]^ Copyright 2020, AAAS.

Wang et al.^[^ ^216^ ^]^ introduced an innovative visualizable nanozyme based on the “unlocking” mechanism of the TME for enhanced combination therapy of breast cancer, highlighting its application in nanozyme PTT. The core idea is to achieve efficient tumor clearance through the specific triggering of the TME and the synergistic effects of PTT and ROS generation, with the ability to visualize tracking in vivo. The nanozyme itself has excellent photothermal conversion capabilities. Under NIR irradiation, the nanozyme can efficiently absorb light energy and convert it into heat energy, thereby achieving local heating and killing of tumor cells. The nanozyme is designed to respond to specific stimuli in the TME to efficiently generate ROS. The generation of ROS further enhances the damage to tumor cells and synergizes with the photothermal effect. The study proposes a synergistic enhancement strategy of “TME unlocking” that combines the therapeutic function of the nanozyme with tumor vascular normalization. This means that nanozymes not only act directly on tumor cells, but also improve drug delivery and therapeutic effects by modulating the TME. The nanozyme also has excellent fluorescence imaging and magnetic resonance imaging (MRI) performance, which allows researchers to visualize and monitor its distribution, accumulation, and therapeutic process in vivo in real time. This multifunctional nanozyme has achieved encouraging results in the treatment of breast cancer. The nanozyme exhibits high photothermal conversion efficiency under NIR light irradiation and can efficiently generate ROS under TME stimulation. Its “high efficiency” and “remarkable” performance are emphasized. The nanozyme exhibits “remarkable imaging performance” in both near‐infrared fluorescence imaging and MRI, supporting its ability as a visual tracer. This work provides important inspiration for nanozyme PTT of tumors. It proves the feasibility of designing multifunctional nanozymes that integrate PTT, ROS generation, and imaging diagnosis. This represents the future direction of tumor treatment development, namely, the synergy of diagnosis and treatment through a single nano platform. It emphasizes the importance of nanozyme response to TME. Through the TME “unlocking” mechanism, nanozymes can accurately exert their effects at the lesion site, reducing off‐target toxicity and improving treatment efficiency. This provides new ideas for the development of more intelligent and safer cancer therapies. The combination of different mechanisms, such as PTT and ROS generation, can produce synergistic antitumor effects and effectively overcome the limitations of single therapies. This multimodal combination therapy is expected to improve the treatment success rate of refractory tumors. (**Figure** **10**)

**Figure 11:**
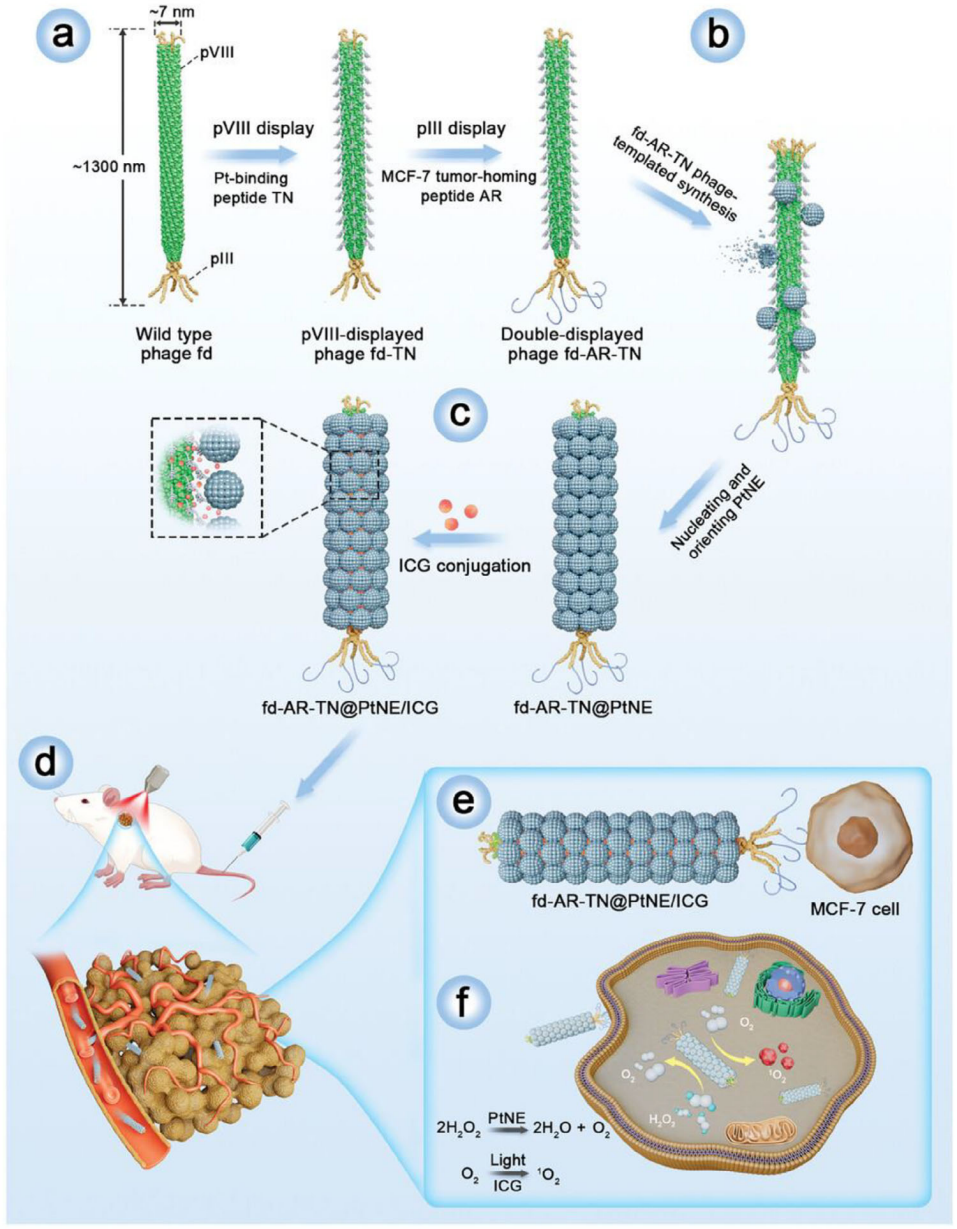
Schematic of Nanozyme‐Enhanced Targeted Breast Cancer Therapy via Dual‐Peptide‐Display Phage Nanofibers Coated with PtNEs. a) Genetic modification of fd phage nanofibers (≈1300 nm long, ≈7 nm wide) to display Pt‐binding peptide (TN) on pVIII and MCF‐7 tumor‐homing peptide (AR) on pIII, producing fd‐AR‐TN. b) Phage‐templated nucleation and oriented assembly of PtNEs on pVIII‐displayed TN of fd‐AR‐TN from Pt precursor solution, forming fd‐AR‐TN@PtNE. c) Covalent conjugation of ICG photosensitizers to free −NH_2_ groups on pVIII of fd‐AR‐TN@PtNE, yielding ICG‐loaded fd‐AR‐TN@PtNE/ICG. d) Intravenous injection of fd‐AR‐TN@PtNE/ICG into MCF‐7 tumor‐bearing mice for therapy. e) Nanofibers target MCF‐7 tumors via AR peptide on phage pIII. f) PtNEs on nanofibers catalyze H_2_O_2_ to generate O_2_ continuously, boosting ROS production under NIR irradiation and enhancing breast cancer PDT by fd‐AR‐TN@PtNE/ICG. Reproduced with permission.^[^ ^210^ ^]^ Copyright 2024, Wiley‐VCH.

Yang et al.^[^ ^210^ ^]^ introduced an innovative tumor‐targeting phage nanofiber for nanozyme‐enhanced targeted PDT for breast cancer. It should be noted that this work focuses on overcoming tumor hypoxia through nanozymes to enhance the effect of PDT, rather than directly performing PTT. Human‐friendly filamentous phages (fd phages) are used as the skeleton of the biological nanofiber. This phage was genetically engineered to display Pt‐binding peptides and tumor‐targeting peptides on its sidewalls and tips, respectively. The tumor‐targeting peptides endowed the nanofibers with the ability to specifically target breast tumors. The Pt‐binding peptides induced Pt nanozymes (PtNEs) to nucleate and arrange themselves in an orderly manner on the sidewalls of the phage nanofibers. These Pt nanozymes exhibit excellent catalase (CAT) mimetic activity and can efficiently catalyze the conversion of high concentrations of H_2_O_2_ into oxygen (O_2_) inside tumors. The nanofibers are also loaded with the photosensitizer indocyanine green (ICG). Under laser irradiation, ICG utilizes the O_2_ generated by PtNEs to efficiently produce ROS, thereby achieving PDT‐mediated killing of cancer cells. Through the oxygen‐producing effect of PtNEs in the TME, hypoxia is effectively alleviated, providing sufficient oxygen substrates for the generation of ROS required for PDT, thereby significantly enhancing the therapeutic efficiency of PDT. PtNEs‐coated tumor‐targeting phage nanofibers exhibit significantly enhanced sustained catalytic activity for converting hydrogen peroxide into O_2_ in hypoxic tumor environments compared to PtNEs without phage templates. Density functional theory calculations also validated the catalytic mechanism of phage‐templated PtNEs. After intravenous injection, the nanofibers efficiently homed to the mammary tumor site, achieving precise targeted drug delivery. In a mouse model of mammary tumors, PtNEs‐enhanced PDT effectively inhibited tumor growth. This means that PDT combined with nanozyme oxygen production can significantly reduce tumor volume or slow its growth. The authors demonstrated that nanozymes can act as “oxygen generators” to effectively overcome hypoxia in solid tumors, which is a major limiting factor in PDT. This provides a reference for other cancer treatment strategies affected by hypoxia such as radiotherapy and certain chemotherapies. The use of phages as genetically engineered biological nanocarriers integrates multiple functions such as tumor targeting, nanozyme loading, and photosensitizer delivery, demonstrating the advantages of a multifunctional integrated nanotherapy platform. The successful combination of biologically sourced phages with inorganic Pt nanozymes has created a highly efficient hybrid nanosystem, expanding the design ideas and application potential of nanozymes in the field of biomedicine. In summary, this study provides a groundbreaking example of how nanozymes can be used to intelligently regulate the TME, particularly to solve the problem of tumor hypoxia, thereby greatly enhancing the effectiveness of PDT against breast cancer. (**Figure** **11**)

### 4.4 Multimodal Synergistic Therapies Based on Nanozyme Platforms

Single therapies such as PTT, PDT, immunotherapy, and CDT have certain limitations in cancer treatment and cannot completely cure persistent tumors. The challenges of recurrence or metastasis still exist, which has led to the development of combination therapy strategies to achieve more effective cancer treatment. Compared to monotherapy, synergistic therapy integrates the advantages of multiple treatment methods into a single nanotechnology‐based system, achieving a synergistic effect where 1+1>2, thereby enhancing anticancer activity and providing a better option for tumor treatment.^[^ ^217^, ^218^, ^219^ ^]^ Phototherapy is effective only for superficial tumors, and tumors are prone to recurrence and metastasis after treatment. Tumor immunotherapy has low treatment efficacy and may cause severe immune toxicity, resulting in suboptimal clinical outcomes. However, immunotherapy can induce systemic immune responses, preventing tumor recurrence and metastasis after phototherapy. Additionally, phototherapy can convert “cold” tumors with low immunogenicity into “hot” tumors with high immunogenicity, thereby enhancing anti‐tumor immune responses and improving anticancer efficacy.^[^ ^220^, ^221^, ^222^ ^]^ Therefore, the combination of phototherapy and immunotherapy can address the limitations of single therapies to some extent, thereby enhancing anticancer efficacy. Phototherapy based on nanomaterials can activate anti‐tumor immune responses through tumor antigens or immune‐related molecules produced by dead cells. However, due to low immune activity, it cannot inhibit the growth of secondary tumors or prevent tumor metastasis. The combined use of photothermal agents with immune checkpoint inhibitors can achieve synergistic effects between phototherapy and immunotherapy, thereby achieving efficient anti‐tumor effects and inhibiting tumor metastasis.

**Figure 12:**
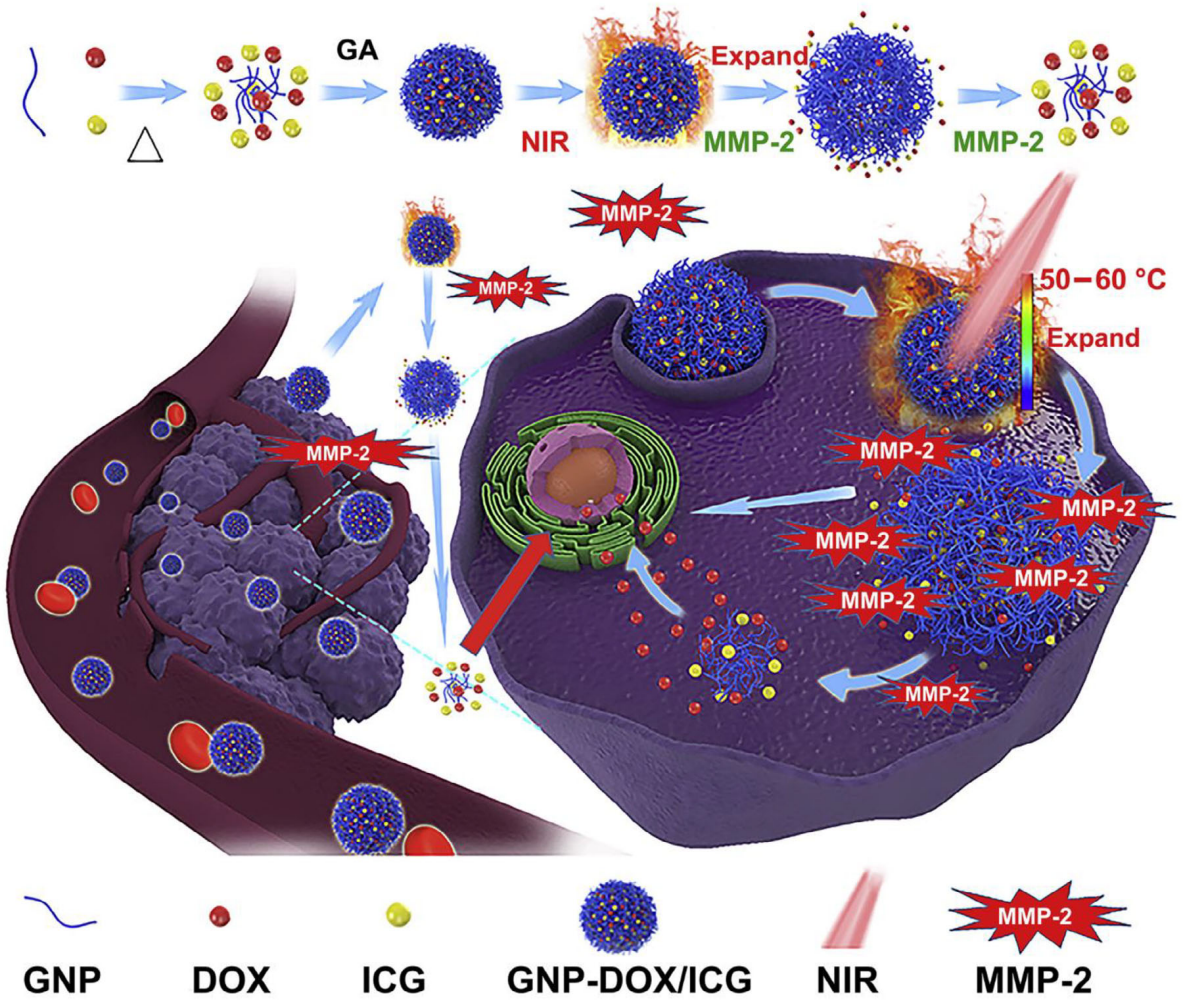
Schematic illustration for GNP‐DOX/ICG preparation and subsequent blood circulation, cellular uptake, and responsive drug release enabled by laser irradiation and MMP‐2 degradation. Reproduced with permission.^[^ ^223^ ^]^ Copyright 2021, Elsevier.

Chen et al.^[^ ^223^ ^]^ introduced a photothermal/matrix metalloproteinase‐2 (MMP‐2) dual‐responsive gelatin nanoparticle (GNP‐DOX/ICG) for chemotherapy‐photothermal combination therapy in breast cancer. This design leverages the thermal sensitivity of gelatin and the overexpression of MMP‐2 in tumors to achieve on‐demand drug release and enhanced antitumor efficacy, with PTT as one of its core mechanisms. By leveraging gelatin's thermal sensitivity, a size‐variable gelatin nanoparticle (GNP) was designed and loaded with the photothermal agent ICG and the chemotherapy drug DOX. Under 808 nm near‐infrared laser irradiation, GNP‐DOX/ICG undergoes photothermal response and expands, facilitating particle retention at the tumor site and release of the loaded therapeutic agents. MMP‐2, which is overexpressed in the TME, can degrade gelatin. Upon reaching the tumor site, the degradative action of MMP‐2 causes the nanoparticles to shrink in size, further promoting drug release and penetration into tumor tissue. By combining PTT with chemotherapy, GNP‐DOX/ICG aims to achieve synergistically enhanced therapeutic effects. The photothermal effect can directly kill tumor cells while promoting the release and cellular uptake of chemotherapy drugs through thermal effects, thereby enhancing overall antitumor efficacy. Nanoparticle size plays a critical role in tumor accumulation and retention. This design optimizes nanoparticle behavior in vivo through laser‐induced expansion and MMP‐2‐induced shrinkage, aiming to enhance accumulation and retention at the tumor site while minimizing side effects on normal tissues. Under 808 nm laser irradiation (1 W/cm^2^), the size of GNP‐DOX/ICG expanded from 71.58 ± 4.28 nm to 160.80 ± 9.51 nm. Under the action of MMP‐2, which is overexpressed in tumors, the size of GNP‐DOX/ICG particles decreased to 33.24 ± 4.11 nm, which further improved drug release. For GNP‐DOX/ICG with high cross‐linking density, the drug loading of DOX was 8.23 ± 0.68%, and the encapsulation efficiency was 18.68 ± 1.42%. The drug loading of ICG was 10.12 ± 0.12%, with an encapsulation efficiency of 78.86 ± 1.01%. GNP‐DOX/ICG effectively inhibited the growth of 4T1 tumors in vivo, demonstrating enhanced synergistic therapeutic effects. This work provides important insights for chemotherapy‐photothermal combined therapy in breast cancer. The successful construction of nanoparticles that respond to both photothermal and enzymatic stimuli provides a new approach to achieving “on‐demand” drug release and improving treatment accuracy. By utilizing the size changes of nanoparticles under different physiological conditions, their accumulation, penetration, and retention in tumor sites can be optimized, which is of great significance for improving tumor treatment efficacy and reducing off‐target toxicity. This study further confirms that PTT not only directly kills tumor cells but also synergizes with chemotherapy drugs by enhancing drug penetration and release, thereby significantly improving overall antitumor efficacy. In summary, this study provides an innovative strategy for a gelatin‐based chemotherapy‐photothermal nanoparticle delivery system, achieving synergistic enhanced therapy for breast cancer through intelligent size and drug release control, offering valuable references for the development of tumor nanomedicine. (**Figure** **12**)

**Figure 13:**
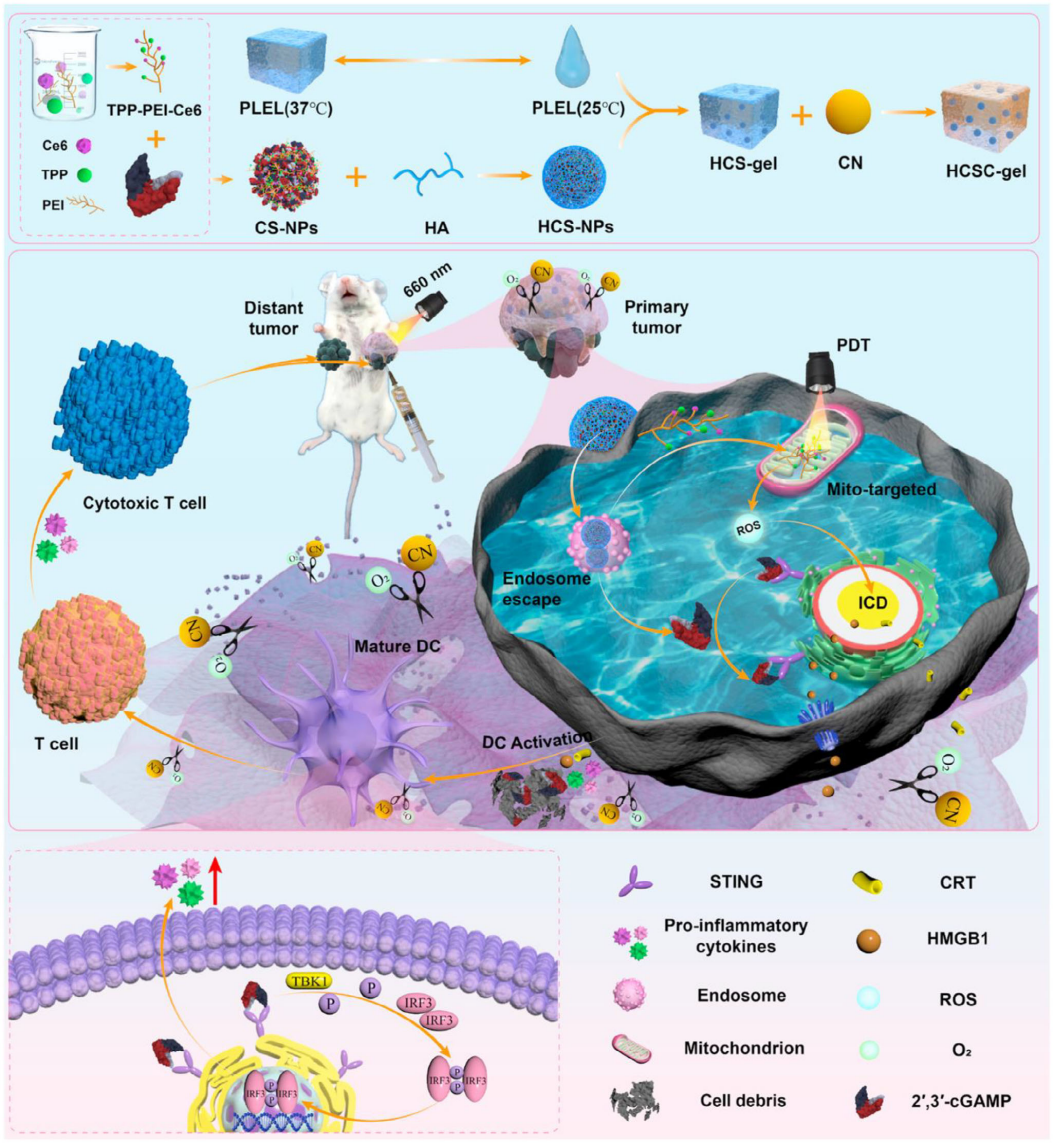
The facile construction of HCSC‐gel and their utilization in combinatorial therapies for breast cancer. Reproduced with permission.^[^ ^224^ ^]^ Copyright 2025, Elsevier.

Yang et al.^[^ ^224^ ^]^ proposed a multifunctional nanogel system (HCSC‐gel) that combines the enzymatic degradation of collagenase (CN), PDT, and STING agonist‐mediated immunotherapy to overcome tumor penetration barriers and immunosuppression in breast cancer treatment and achieve synergistic treatment of primary and metastatic lesions. This work offers an innovative strategy for the transformation and efficient treatment of “cold” tumors from the perspective of the combined mechanism of nanomaterials and enzymes. After injection into the primary tumor site, HCSC‐gel rapidly forms a gel matrix and locally releases CN. As an enzyme, CN can efficiently degrade the dense ECM of tumors, thereby significantly enhancing the penetration depth and efficiency of photosensitizers, STING agonists, and oxygen into tumor tissues. This enzymatic action is a key step in enabling the nanosystem to penetrate deep into tumors and exert its effects. The system co‐loads a mitochondrial‐targeted photosensitizer (TPP‐PEI‐Ce6). Under laser irradiation, the photosensitizer efficiently generates ROS in mitochondria‐rich regions, inducing photodynamic damage and ICD in tumor cells. The HCSC‐gel simultaneously delivers the STING agonist (2',3'‐cGAMP). Activation of the STING pathway converts low‐immunogenic “cold” tumors into high‐immunogenic “hot” tumors, thereby inducing a robust anti‐tumor immune response and effectively treating primary and distant metastatic lesions. The HCSC‐gel system itself is a thermosensitive hydrogel that rapidly forms a gel at the tumor site after injection, enabling local retention and controlled release of the therapeutic agent, ensuring effective action time of the components in the TME. The synthesis yield of the mitochondrial‐targeted photosensitizer TPP‐PEI‐Ce6 reached 70.5%, with a grafting efficiency of ≈31.6% for Ce6. The self‐assembled HCS‐NPs exhibit a uniform spherical morphology, with an average hydrated particle size of 154.28 ± 10.21 nm and a Zeta potential of ‐(18.8 ± 3.41) mV. HCS‐NPs significantly enhanced the mitochondrial targeting effect on 4T1 cells. Compared with the free Ce6 group, the average Pearson correlation coefficients for the free Ce6, TPP‐PEI‐Ce6, and HCS‐NPs groups were 0.68, 0.87, and 0.96, respectively, indicating that HCS‐NPs exhibit the highest mitochondrial colocalization efficiency. The intracellular ROS production in the TPP‐PEI‐Ce6 and HCS‐NPs groups was significantly stronger than in the free Ce6 group, with the HCS‐NPs group primarily promoting PDT through the generation of singlet oxygen (^1^O_2_). HCS‐NPs significantly enhance the cellular uptake efficiency of Ce6. The HCSC‐gel system exhibits phase transition characteristics at ≈37°C and 45°C, maintains good fluidity at 4°C, and undergoes a sol‐gel transition at 37°C, consistent with its thermoresponsive properties. CN is released from the hydrogel earlier than TPP‐PEI‐Ce6, which facilitates the premature disruption of the ECM and promotes the deep penetration of subsequent photosensitizers. The HCSC‐gel system effectively activates “cold” tumor immunity and effectively treats primary and metastatic lesions. This work provides important insights for the treatment of breast cancer. The successful combination of enzymatic degradation, PDT, and immunotherapy in a single nanodelivery platform specifically addresses the challenges of dense tumor ECM barriers and immunosuppression. By inducing ICD through PDT and activating immune responses with STING agonists, the system achieves the transformation of low‐immunogenic tumors into high‐immunogenic tumors, opening new avenues for improving treatment outcomes for tumors resistant to traditional immunotherapy. The local gelation and controlled release properties of thermosensitive hydrogels, combined with enzymatic degradation, ensure efficient accumulation and deep penetration of therapeutic agents at tumor sites, enhancing treatment precision and safety. (**Figure** **13**)

**Figure 14:**
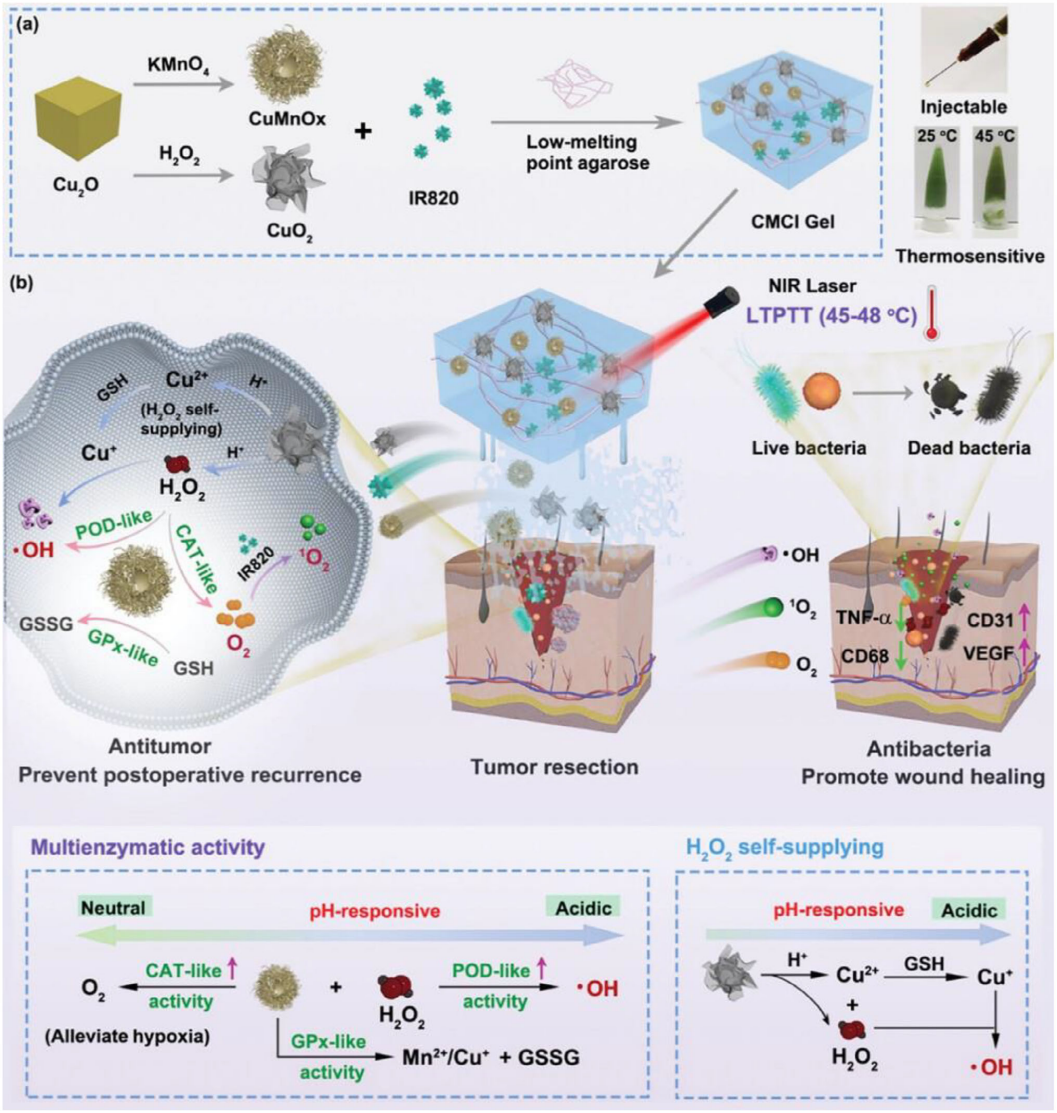
Schematic diagram showing (a) the preparation process of CMCI Gel and (b) its therapeutic principle as well as potential application scenarios. This gel is capable of suppressing postoperative recurrence and wound infection. Reproduced with permission.^[^ ^225^ ^]^ Copyright 2025, Wiley‐VCH.

Hu et al.^[^ ^225^ ^]^ developed a novel microenvironment‐responsive injectable thermosensitive hydrogel that integrates multiple nanozymes for the synergistic treatment and postoperative adjuvant therapy of breast cancer. This work demonstrates the great potential of multifunctional nanozymes in precision tumor treatment and postoperative complication management from the perspective of the combined mechanism of nanozymes. The system contains two key nanozymes. Honeycomb CuMnOx nanozyme: has multi‐enzyme activity and pH‐responsive properties. Its enzyme activity can be regulated by pH changes in the tumor or wound microenvironment. CuO_2_ nanoflowers: can overcome the limitation of endogenous H_2_O_2_ deficiency and release ROS under weakly acidic conditions. At the same time, CuO_2_ nanoflowers can also consume glucose, thereby promoting starvation therapy. Nanozymes and the photothermal agent IR820 are co‐encapsulated in a thermosensitive hydrogel. Under NIR irradiation, IR820 can generate mild heat (below 48°C). This mild photothermal effect not only directly damages cancer cells, but also synergistically enhances the catalytic activity of nanozymes and the generation of ROS, thereby improving the overall treatment efficiency. Nanozymes and photothermal agents are co‐assembled into a low‐melting‐point agarose solution to form a thermosensitive nanozyme‐based hydrogel (CuMnOx@CuO_2_@IR820 hydrogel, abbreviated as CMCI Gel). After injection, the gel rapidly forms at the tumor site, achieving controlled release and local enrichment of the therapeutic agent, providing a stable microenvironment for synergistic treatment. CMCI Gel achieves multimodal attack on tumors by combining nanozyme‐mediated ROS generation, glucose consumption, and mild photothermal effects. In addition, the system is also designed for postoperative adjuvant therapy, aiming to prevent wound infection through its antibacterial activity and inhibit tumor recurrence through its sustained antitumor effect. The average particle size of the honeycomb CuMnOx nanozyme is 31.02 ± 3.49 nm. The leaf thickness of CuO_2_ nanoflowers is ≈3.31 ± 0.61 nm. Under 808 nm laser irradiation (1 Wcm^−^^2^), CMCI Gel can reach a temperature of 46.8 °C within 10 min, confirming its effective hypothermic photothermal capability. CMCI Gel can significantly consume glucose, consuming 81.5% of glucose within 24 h, indicating its potential in starvation therapy. In in vivo experiments, CMCI Gel combined with laser treatment can efficiently inhibit primary tumor growth and lung metastasis, with a tumor growth inhibition rate of 94.1% and a lung metastasis inhibition rate of 90.7%. CMCI Gel showed significant antibacterial activity against Staphylococcus aureus and Escherichia coli, proving its potential in preventing postoperative infections. This study provides inspiration for the application of nanozymes in tumor treatment and postoperative management. The successful integration of multiple enzyme‐mimetic activities and photothermal effects has achieved a combined attack on tumors, providing an effective strategy to overcome the limitations of single therapies. The pH and temperature responsiveness have enabled precise release and activity regulation of the therapeutic agent, improving the targeting and safety of the treatment. Through the innovative design of a combined nanozyme mechanism, this study demonstrates the enormous potential of smart nanoplatforms in solving the complex challenges of cancer treatment. (**Figure** **14**)

## 5 Challenges and Clinical Translation Barriers

Despite rapid progress in preclinical studies, the clinical translation of nanozymes for breast cancer therapy remains highly challenging. Key obstacles include limited biocompatibility evaluation, insufficient understanding of in vivo pharmacokinetics, and inefficient delivery to deep tumor sites. Moreover, tumor heterogeneity, an immunosuppressive microenvironment, and off‐target accumulation further restrict therapeutic efficacy.^[^ ^226^, ^227^, ^228^, ^229^ ^]^ Beyond biological barriers, translational progress is constrained by difficulties in large‐scale and reproducible synthesis, quality control, and long‐term safety assessment. Regulatory approval pathways for nanozymes are still underdeveloped, requiring standardized protocols for characterization, toxicity evaluation, and biosafety monitoring. Ethical considerations, cost of production, and accessibility for patients also add complexity to future clinical implementation.^[^ ^230^, ^231^, ^232^ ^]^ To address these challenges, future research should prioritize the development of tumor‐microenvironment–responsive designs, strategies to enhance systemic delivery and tumor penetration, and multifunctional platforms capable of precise spatiotemporal control. Equally important are efforts to establish standardized evaluation frameworks and interdisciplinary collaborations to accelerate regulatory approval. Only by comprehensively addressing these barriers can the therapeutic promise of nanozymes be effectively translated into clinical practice for breast cancer patients.^[^ ^233^, ^234^, ^235^ ^]^

### 5.1 In Vivo Metabolism, Degradation Pathways, and Long‐Term Toxicity Issues

Gaining a thorough understanding of the in vivo metabolism and degradation pathways of nanozymes in complex biological environments is a key challenge for their clinical translation. Their degradation products and pathways must be thoroughly investigated to ensure that they do not accumulate in organs or tissues or produce toxic metabolites that could cause damage. Currently, little is known about the long‐term effects of nanozymes in the body. Existing preclinical studies typically provide only short‐term biocompatibility data, which is insufficient for assessing potential chronic toxicity. In particular, for single‐atom nanozymes (SAzymes), although they perform well in terms of metal efficiency optimization, their long‐term toxicity, clearance pathways, and bioaccumulation characteristics have not been fully explored.^[^ ^236^ ^]^ This raises concerns about the accumulation of persistent nanomaterials in organs within the body and the possibility of unpredictable toxic effects. In addition, the environmental impact of nanozyme disposal is also a growing concern.^[^ ^237^ ^]^ Certain heavy metal‐based nanozymes may be absorbed by soil and water, causing ecological pollution and ultimately harming human health through bioaccumulation in the food chain.^[^ ^238^ ^]^ Certain carbon‐based nanomaterials, such as graphene and quantum dots, also possess inherent toxicity.^[^ ^239^ ^]^ Drawing from historical experience in the development of new technologies, the long‐term impacts of nanotechnology on human health and the environment are critical areas requiring urgent research and reflection.

### 5.2 Immunogenicity: Immune Response Mechanisms and Avoidance Strategies

As a new type of synthetic material, nanozymes pose a risk of triggering adverse immune responses in the body.^[^ ^240^ ^]^ This immunogenicity may lead to adverse reactions, reduced therapeutic effects, or rapid clearance by the circulatory system, thereby seriously hindering their clinical application. Nanocarriers can trigger a variety of immune responses, including inflammation, allergic reactions, or other systemic effects. Following intravenous administration, nanoparticles inevitably interact with biological fluids, leading to the adsorption of biomolecules onto their surfaces, forming a “protein corona.^[^ ^241^ ^]^” This protein crown alters the surface properties of nanozymes, leading to recognition by the immune system and accelerated clearance by the reticuloendothelial system (RES).^[^ ^242^ ^]^ In severe cases, excessive immune activation can lead to systemic inflammatory responses such as cytokine release syndrome. To reduce immunogenicity, it is essential to understand the interaction between nanozymes and immune cells. Surface modification is a key strategy for masking nanoparticles from immune system recognition and enhancing biocompatibility. For example, surface functionalization using biocompatible polymers such as polyethylene glycol is a widely explored cutting‐edge strategy that can improve stability, reduce toxicity, and decrease immunogenicity.^[^ ^243^ ^]^ Bionic approaches, such as coating nanozymes with red blood cell membranes or tumor‐derived exosome membranes, can confer “self‐recognition” properties, thereby achieving immune escape and prolonging circulation time, while also potentially providing homogenous targeting capabilities.^[^ ^244^ ^]^

### 5.3 Strategies for Enhancing Biocompatibility: Surface Modification and Biodegradable Platforms

Biocompatibility, defined as the ability of a material to elicit an appropriate host response when implanted into a biological system, is a prerequisite for nanozyme applications. The chemical composition and structural characteristics of nanozymes significantly affect their interaction with biological systems. Biodegradable materials, such as polylactic‐co‐glycolic acid or carbon dots, are generally more biocompatible than metal nanoparticles that may release toxic metal ions.^[^ ^245^ ^]^ Therefore, the priority development of biodegradable nanozyme platforms is essential for safe clinical translation. Surface modification with biocompatible polymers, peptides, or antibodies can improve the stability of nanozymes, reduce toxicity, and enhance targeting efficiency.^[^ ^246^ ^]^ Comprehensive in vitro and in vivo testing is essential for assessing the biocompatibility and safety of nanozymes prior to clinical application.^[^ ^247^ ^]^ These studies should evaluate biodistribution, pharmacokinetics, and potential long‐term effects. To detect subtle immunotoxicity effects that may be missed by traditional testing methods, advanced analytical techniques such as multi‐omics analysis are essential for robust safety assessment and clinical translation. A key strategy for mitigating safety concerns is to consider and introduce nanomaterials with improved biocompatibility and biodegradability from the design stage. Currently, the paradigm for nanozyme safety assessment is undergoing an important evolution. The emphasis on biodegradable platforms and multi‐omics analysis indicates that the field is shifting from simply observing macrotoxicological endpoints to a more complex, mechanistic, molecular‐level understanding of nanozyme‐biological interactions. This deeper understanding is critical for predicting and mitigating subtle, long‐term adverse effects that may be overlooked by traditional toxicology. However, the biocompatibility of nanozymes faces a dynamic and complex challenge. Although surface modifications such as PEGylation and biomimicry are widely regarded as effective strategies for immune escape and prolonged circulation time, nanoparticles inevitably form a “protein corona” upon contact with biological fluids. This protein corona can significantly alter the properties of nanozymes, including toxicity, cellular uptake, and targeting characteristics, and may even lead to aggregation and inactivation, thereby negating the intended benefits of these surface modifications. This highlights the complexity of precisely controlling nanozyme behavior in vivo, requiring more sophisticated strategies, such as engineering “hard” or “soft” protein coats to achieve controlled intracellular activation, rather than simply preventing the formation of protein coats.^[^ ^248^ ^]^

### 5.4 Targeting Efficiency and In Vivo Distribution: Achieving Precise Delivery

The effectiveness of nanozymes in breast cancer treatment largely depends on their ability to efficiently and specifically reach and penetrate the tumor site while avoiding non‐specific accumulation in healthy tissue. Tumor tissue typically has an immature and porous vascular system, which provides a pathway for circulating particles to enter, known as the enhanced permeability and retention (EPR) effect.^[^ ^249^ ^]^ Although the EPR effect was identified decades ago and initially regarded as a major mechanism for nanoparticle accumulation in tumors, recent clinical studies have demonstrated that its contribution in humans is negligible. Increasing evidence suggests that the previously observed “EPR effect” may be more accurately explained by alternative mechanisms, including active transcytosis, vascular modulation, and ligand‐directed targeting, rather than passive leakage alone.^[^ ^250^ ^]^ Additionally, the dense ECM within tumors contains high levels of collagen, significantly hindering the diffusion of nanoparticle carriers into deeper tumor regions. This constitutes a significant physical barrier to nanoparticle penetration. Elevated IFP within tumors further impedes nanoparticle penetration. The conflicting size requirements for nanoparticles also pose challenges: larger sizes (60–100 nm) are advantageous for prolonging circulation time and enhancing the EPR effect, while smaller sizes (<30 nm, ideally <10 nm) are critical for deep tumor penetration. Intravenously administered nanodrugs inevitably adsorb plasma proteins in vivo, accelerating their recognition and phagocytosis by the reticuloendothelial system (RES), leading to rapid drug clearance and making it difficult for most nanodrugs to effectively reach tumor sites. The half‐life of nanoparticles in blood is influenced by their size and surface chemistry; generally, smaller‐sized nanoparticles and higher‐molecular‐weight mPEG contribute to extended half‐lives.^[^ ^251^ ^]^ Many nanomaterials have low immune escape capabilities and are easily cleared by organs such as the liver and kidneys. To overcome these challenges, researchers have developed a variety of strategies: Active targeting: By functionalizing nanozymes with specific targeting ligands, precise delivery and controlled release to diseased tissues can be achieved, thereby minimizing side effects.^[^ ^252^ ^]^ For example, HER2‐targeted peptides can actively recognize and target breast cancer cells.^[^ ^253^ ^]^ Biomimetic strategies, such as macrophage membrane or exosome coating, can enhance targeting and prolong blood retention time.^[^ ^254^ ^]^ Responsive size switching: This strategy aims to design nanoparticles that remain large in size during circulation and switch to a smaller size upon arrival at the tumor site through specific stimuli.^[^ ^255^ ^]^ Examples include MMP‐2‐degradable gelatin‐based nanoparticles or pH‐responsive cluster nanoparticle systems that disintegrate into smaller particles in an acidic TME.^[^ ^256^ ^]^ ECM degradation: Functionally engineering enzymes that degrade ECM components onto nanoparticles can enhance tumor penetration and improve drug distribution.^[^ ^257^ ^]^ However, it should be noted that ECM degradation may also promote tumor cell migration and metastasis.^[^ ^258^ ^]^ Physicochemical property optimization: The size, shape, and surface charge of nanoparticles have a profound impact on their tumor penetration ability.^[^ ^259^, ^260^ ^]^ Particles below 100 nm are typically most suitable for the EPR effect. Compared to larger sizes, smaller nanoparticles exhibit higher penetration rates within tumors. Surface properties such as surface charge and hydrophilicity/hydrophobicity also influence cellular uptake and biodistribution. The interplay between nanoparticle size, mPEG molecular weight, blood half‐life, and tumor accumulation/penetration reveals a complex optimization problem. There is a trade‐off between maximizing accumulation, typically achieved by larger particles within the EPR effect window, and achieving deep penetration. This highlights the necessity of multi‐level delivery systems or size‐switching strategies to reconcile these conflicting requirements. Additionally, while ECM degradation strategies can enhance tumor penetration, the accompanying risk is that such degradation may inadvertently promote tumor cell migration and metastasis. This is not only a technical challenge but also a critical safety issue and complex ethical dilemma that requires careful consideration in clinical translation.

### 5.5 Large‐Scale Production and Quality Control

To move from laboratory research to clinical application, nanozymes must overcome significant obstacles in terms of large‐scale production, quality control, and economic feasibility. Achieving consistency in the large‐scale production of nanozymes and ensuring uniformity in size, catalytic activity, and surface properties is a daunting challenge.^[^ ^261^, ^262^, ^263^ ^]^ Any slight difference can affect therapeutic efficacy and biosafety. Although some nanozymes are relatively easy to synthesize, other complex nanozyme systems involve multi‐step procedures that are difficult to scale up without compromising reproducibility. In addition, large‐scale production of complex nanozyme carrier systems may involve expensive materials, energy‐intensive processes, and high‐cost facilities, making cost‐effective manufacturing extremely challenging.^[^ ^264^, ^265^, ^266^ ^]^ The transition from laboratory‐scale synthesis to commercial production requires ensuring that each system meets strict quality standards. To ensure the purity, efficacy, and safety of nanozyme products, robust quality control measures must be established to meet the stringent requirements of drug production. Standardized testing protocols should be developed to ensure repeatability and reliability. Currently, there is a lack of standardized protocols for the synthesis, testing, and validation of nanozyme‐based detection methods, which hinders their wider application. Specific protocols for measuring and defining catalytic activity units and kinetics are being developed to enable quantitative comparisons and facilitate development. Advanced analytical techniques are essential for the comprehensive characterization of nanozymes to confirm successful synthesis and properties. Manufacturers must adhere to current Good Manufacturing Practice guidelines, thoroughly describe critical quality attributes, and rigorously assess safety, efficacy, and quality. Common stability issues with nanomaterials in drug products include changes in size and/or size distribution, changes in morphology or solid state, or aggregation/agglomeration. Economic feasibility through cost‐effective manufacturing is critical to the widespread accessibility of nanozymes in clinical applications. Although the production cost of nanozymes is generally lower than that of natural enzymes, the complexity of advanced nanozyme systems can drive up costs. The global nanotechnology market is growing significantly, with biotechnology applications representing a high‐cost, low‐volume market requiring more complex and functionalized particles.^[^ ^267^, ^268^, ^269^ ^]^ The initial investment for a nano‐magnetic particle factory for biotechnology applications can be considerable, but the potential profitability is also high. The challenge of “repeatability” of nanozymes is directly related to the need for “standardized testing methods” and the application of “advanced manufacturing technologies”. Without robust quality control and scalable precision manufacturing, reproducibility will be a major barrier to clinical translation, regardless of the therapeutic potential of nanozymes.^[^ ^270^, ^271^, ^272^ ^]^ This suggests that the clinical application of nanozymes requires the active adoption of advanced manufacturing processes guided by standardized quality control. In addition, although cost‐effectiveness is a basic requirement for the widespread application of nanozymes, if nanozymes can address unmet medical needs that are not adequately addressed by existing treatment options, their high cost may be acceptable in the early stages. This provides a strategic entry point for early nanozyme therapies to enter the market, even if their production costs are high. In addition to scientific and technological barriers, the clinical translation of nanozymes also requires careful consideration of regulatory science and approval pathways. Unlike conventional small‐molecule drugs or biologics, nanozyme‐based systems involve complex compositions, multifunctional properties, and dynamic interactions with the tumor microenvironment, which complicate regulatory evaluation. Current approval frameworks for nanomedicines are still evolving, and standardized guidelines for characterization, toxicity assessment, pharmacokinetics, and long‐term biosafety are lacking. Agencies such as the FDA and EMA increasingly emphasize quality‐by‐design approaches, reproducibility of large‐scale production, and robust preclinical models that closely mimic human biology. Therefore, establishing harmonized international standards, developing specialized regulatory evaluation methods, and engaging in early dialogue with regulatory bodies are essential steps to accelerate the clinical translation of nanozymes for breast cancer therapy.

## 6 Conclusion and Future Perspectives

Nanozymes have emerged as a promising and versatile class of therapeutic agents for breast cancer, offering a range of advantages over traditional modalities. Their enzyme‐mimicking catalytic activity, structural tunability, and multifunctional integration make them particularly well‐suited for overcoming challenges such as multidrug resistance, tumor heterogeneity, and systemic toxicity. By modulating the tumor microenvironment, enhancing immunogenicity, enabling controlled drug release, and supporting synergistic therapeutic combinations, nanozymes have demonstrated significant potential to reshape breast cancer treatment paradigms. The development of nanozyme platforms capable of combining phototherapy, chemotherapy, and immunotherapy within a single system underscores their value in enabling precision and personalized medicine.

Nonetheless, several barriers continue to limit the clinical translation of nanozymes. These include difficulties in achieving precise catalytic control under physiological conditions, suboptimal tumor‐targeting efficiency, limited long‐term safety data, and the lack of standardized manufacturing and evaluation protocols. Addressing these challenges will require advances in rational nanozyme design guided by computational chemistry and machine learning approaches, enabling precise engineering of catalytic active sites, improved specificity, and predictable pharmacokinetics. The incorporation of stimulus‐responsive elements, such as pH‐ or enzyme‐sensitive domains, offers the potential for spatiotemporally controlled activation, which is critical for minimizing off‐target effects and maximizing therapeutic efficacy. In parallel, intelligent delivery strategies that exploit tumor‐specific cues to modulate particle size or surface properties in situ are essential for improving deep tissue penetration and overcoming the physical barriers imposed by the tumor microenvironment. Scalable and reproducible production remains a prerequisite for clinical application. This necessitates investment in advanced manufacturing technologies, the establishment of rigorous quality control standards, and the implementation of harmonized characterization methods to ensure inter‐batch consistency and cost‐effectiveness. Regulatory considerations must be addressed early in the development process through proactive dialogue with regulatory agencies, allowing alignment with emerging guidance on nanotherapeutics and the incorporation of adaptive clinical trial designs that leverage biomarker‐based patient stratification. Equally important is the integration of ethical and societal considerations throughout the research and development pipeline. This includes ensuring informed consent, protecting patient privacy, promoting equitable access to nanozyme‐based therapies, and evaluating the environmental impact of large‐scale production and disposal. Another critical area requiring attention is the current lack of long‐term in vivo data. Many nanozyme platforms have demonstrated impressive preclinical efficacy; however, the absence of extended safety studies undermines confidence in their translational readiness. Future research must shift from proof‐of‐concept novelty toward robust validation, including long‐duration pharmacodynamic and pharmacokinetic studies supported by multi‐omics analyses to elucidate metabolism, biodistribution, and immunotoxicity profiles. The development of companion diagnostic technologies that enable real‐time imaging and therapeutic monitoring will further enhance the clinical utility of nanozymes and facilitate their incorporation into precision oncology workflows.

Nanozymes have emerged as a versatile and promising class of therapeutic agents, poised to revolutionize the treatment of breast cancer by overcoming the persistent challenges of multidrug resistance, tumor heterogeneity, and systemic toxicity associated with traditional modalities. However, translating this immense potential into clinical reality requires a concerted and multifaceted effort to navigate the significant barriers ahead. Future progress must pivot from empirical discovery toward the rational design of nanozymes, guided by computational chemistry and machine learning, to achieve precise catalytic control and predictable pharmacokinetics, while incorporating stimulus‐responsive elements for spatiotemporally controlled activation.^[^ ^273^ ^]^ This must be coupled with the development of intelligent delivery strategies that can actively overcome the physical barriers of the tumor microenvironment to ensure deep tissue penetration. Bridging the translational gap also necessitates the establishment of scalable, reproducible manufacturing technologies and rigorous quality control standards to ensure inter‐batch consistency and cost‐effectiveness. Critically, the current lack of long‐term in vivo safety and pharmacokinetic data must be addressed through robust, extended preclinical studies, supported by multi‐omics analyses and the development of companion diagnostics for real‐time therapeutic monitoring. Ultimately, accelerating the clinical translation of nanozymes will depend on fostering an interdisciplinary ecosystem of collaboration between academia, industry, and regulatory agencies, embedding ethical considerations and proactive regulatory dialogue at every stage. Through this holistic, application‐oriented paradigm, nanozymes hold the potential to deliver more accurate, effective, and less toxic interventions, fundamentally improving the quality of life and survival outcomes for breast cancer patients worldwide. Taken together, the field of nanozyme‐based cancer therapy is rapidly evolving from isolated experimental studies toward a more holistic and application‐oriented paradigm. By fostering interdisciplinary collaboration across academia, industry, and regulatory sectors, and by embedding ethical foresight and translational planning at every stage, nanozymes are poised to revolutionize breast cancer treatment. With continued investment and coordinated effort, they hold the potential to deliver more accurate, effective, and less toxic interventions, ultimately improving the quality of life and survival outcomes for patients facing this prevalent and devastating disease.

## Conflict of Interest

The authors declare no conflict of interest.

## Data Availability

No data was used for the research described in the article.
